# Synthesis and Characterization of Phenylboronic Acid-Modified Insulin With Glucose-Dependent Solubility

**DOI:** 10.3389/fchem.2022.859133

**Published:** 2022-03-16

**Authors:** Nai-Pin Lin, Nan Zheng, Landa Purushottam, Yi Wolf Zhang, Danny Hung-Chieh Chou

**Affiliations:** ^1^ Department of Pediatrics, Division of Diabetes and Endocrinology, Stanford University, Stanford, CA, United States; ^2^ Department of Biochemistry, University of Utah, Salt Lake City, UT, United States

**Keywords:** insulin, glucose responsiveness, peptide, insulin modification, phenylboronate

## Abstract

Glucose-responsive insulin represents a promising approach to regulate blood glucose levels. We previously showed that attaching two fluorophenylboronic acid (FPBA) residues to the C-terminal B chain of insulin glargine led to glucose-dependent solubility. Herein, we demonstrated that relocating FPBA from B chain to A chain increased the baseline solubility without affecting its potency. Furthermore, increasing the number of FPBA groups led to increased glucose-dependent solubility.

## 1 Introduction

Insulin therapy is essential to the treatment of type I diabetes (T1D) and some type II diabetes (T2D) (Berenson et al., 2011). These diseases present a complete or partial loss of insulin response or insulin sensitivity, resulting in dysregulation of blood glucose levels. Glycemic control currently relies on an accurate dose of insulin drugs according to the measurement of blood glucose and individual response (DeWitt and Hirsch, 2003). All currently US Food and Drug Administration (FDA)-approved insulin still have a narrow therapeutic window: insulin overdoses lead to hypoglycemia and underdoses result in hyperglycemia (Peyrot et al., 2012). Chronic hyperglycemia can lead to cardiovascular diseases, nephropathy, non-healing wounds, and other diabetic complications, whereas hypoglycemia can result in acute coma or even death (McCoy et al., 2012; Frier, 2014). This challenge of optimal glycemic control remains to be addressed. Glucose-responsive insulin (GRI) derivatives have been developed to address this challenge by making the insulin conjugate itself with a higher glucose-lowering effect in response to elevated blood glucose levels (Veiseh et al., 2015; Bakh et al., 2017; Rege et al., 2017; Disotuar et al., 2020; Jarosinski et al., 2021). To date, carbohydrates (Brownlee and Cerami, 1979; Wang et al., 2017; Kaarsholm et al., 2018), hydrazones (Mannerstedt et al., 2021), and phenylboronic acids (PBA) (Hoeg-Jensen et al., 2005a; Hoeg-Jensen et al., 2005b; Chou et al., 2015; Qiu et al., 2019; Chen et al., 2021) have been conjugated to insulin to achieve glucose responsiveness through different mechanisms.

**FIGURE 1:**
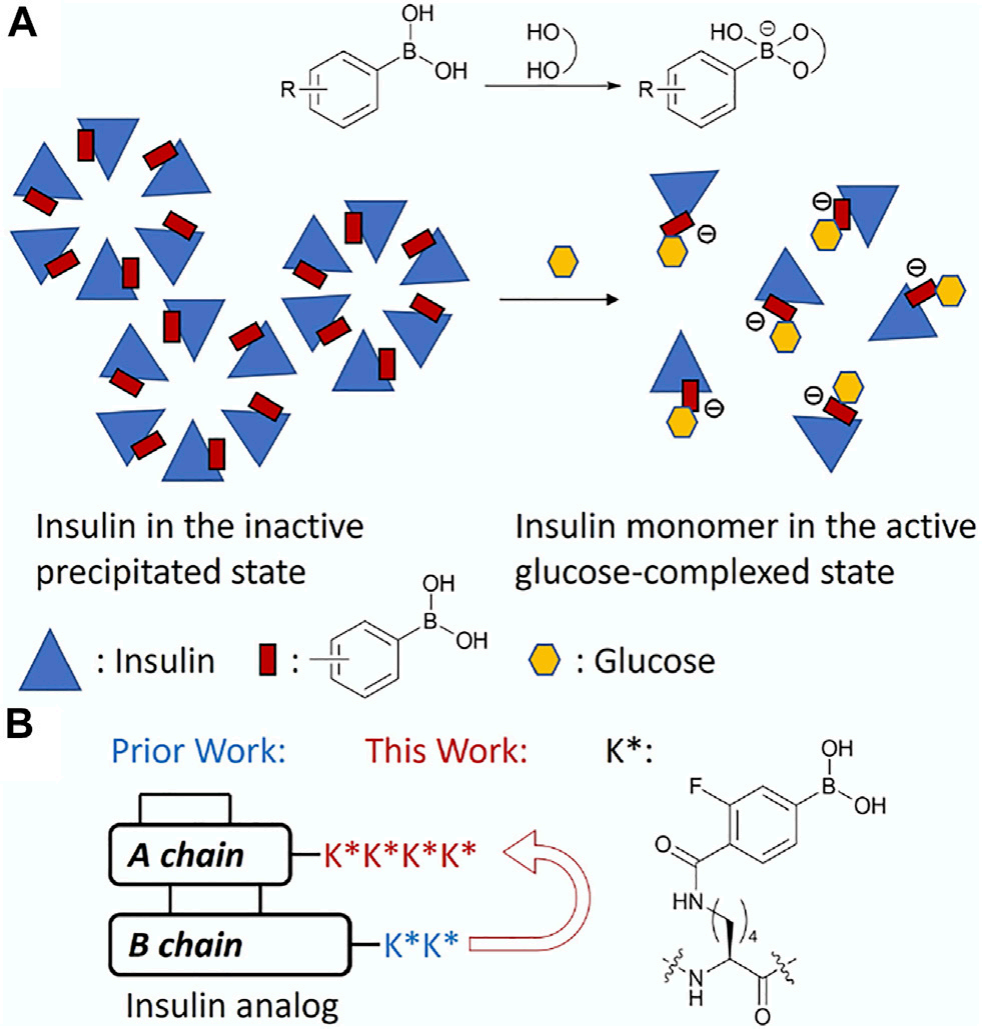
**(A)** Schematic mechanism of glucose-responsive insulin. **(B)** Design of FPBA-conjugated glucose-responsive insulin.

Insulin glargine, marketed as Lantus^®^, is a long-acting insulin analog with a 24-h dosing regimen. The additional 2 arginine residues on the C-terminus of the B chain increases its isoelectric point (pI) 6.7, which lowers its solubility at physiological pH and leads to precipitation at the subcutaneous injection site (Kohn et al., 2007). Glargine is then released slowly from the precipitate into the bloodstream to establish the long-acting property. We previously hypothesized that this releasing mechanism can also be controlled by the glucose concentration to create a GRI, which had a low solubility under a low glucose environment, and the solubility could increase along with the elevating glucose concentration to release more insulin into the bloodstream. PBA is known to reversibly bind to 1,2- and 1,3-diols including glucose. After binding with a diol, PBA is further negatively charged (Furikado et al., 2014). If insulin is conjugated with PBA, the acquired negative charges after glucose binding can lower the pI away from 7.4 resulting in solubility increase and insulin release to bloodstream. The addition to hydrophilic sugar molecules would also increase overall solubility (Figure 1A). We previously reported an insulin analog with two fluorophenylboronic acid (FPBA)-conjugated lysine on the ε-amino group (K*) in the C-terminal B chain with glucose responsiveness through solubility change (Qiu et al., 2019). The electro-withdrawing fluoro substituent can lower the pKa of non-substituted PBA (8.8) to facilitate binding of glucose at physiologic pH (Yan et al., 2004). In this work, we further explore the impacts of the position and numbers of FPBA on glucose-dependent solubility of insulin to optimize the glucose responsiveness (Figure 1B).

## 2 Results and Discussion

**SCHEME 1:**
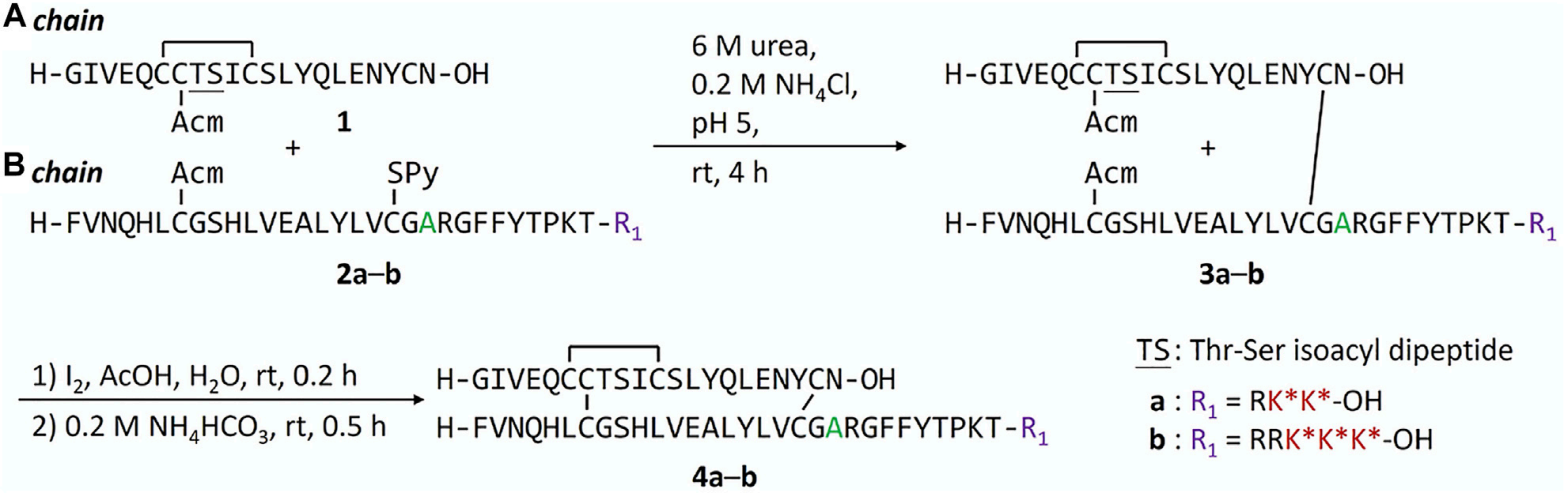
Synthesis of FPBA-conjugated insulin analogs.

**FIGURE 2:**
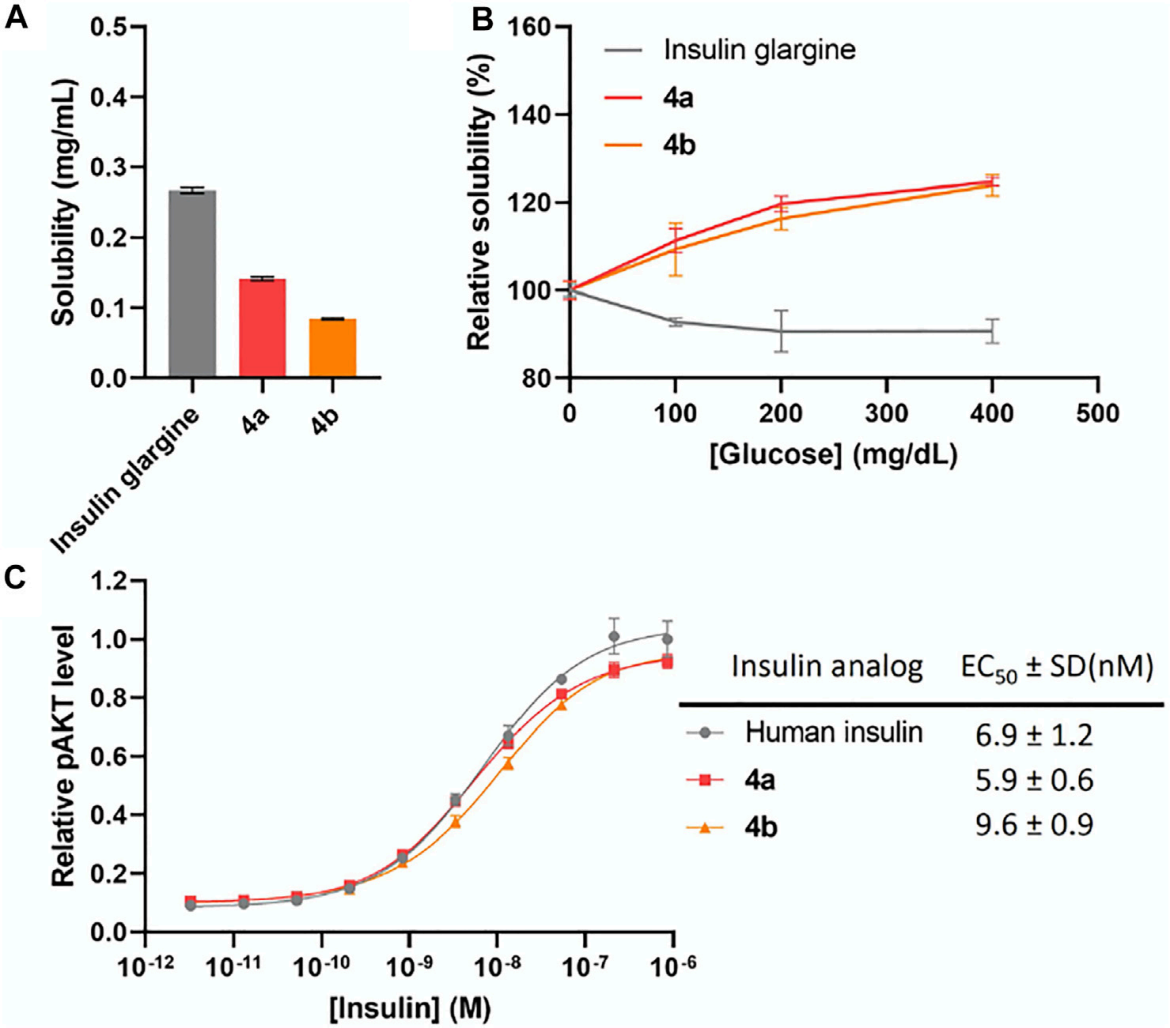
**(A)** Solubility of insulin analogs in pH 7.4 PBS. Data are expressed as mean ± SD (*n* = 4 per group). **(B)** Solubility of insulin analogs in pH 7.4 PBS with different glucose concentrations. Data are expressed as mean ± SD (*n* = 4 per group). **(C)** *In vitro* activity of insulin analogs by using activated pAKT levels as measurements. Data are expressed as mean ± SD (*n* = 4 per group). EC_50_ was calculated by Prism 9 (GraphPad Software, CA, United States) with nonlinear regression curve fitting of dose-response asymmetric equation.

We previously found that addition of FPBA on insulin B chain dramatically decreases the solubility of peptide intermediates and final insulin analogs due to its hydrophobicity (Qiu et al., 2019). During the attempt on the synthesis of human insulin B chain sequence with three FPBA, the solubility of the resulting B chain is too low for purification and subsequent reactions. To circumvent this challenge, we shifted the pI by introducing a Glu to Ala mutation at B21, which was previously shown to have similar insulin receptor affinity (Kristensen et al., 1997). As a result, two additional Arg residues with B21Ala mutation can be synthesized to allow the preparation of the insulin B chain with 3 FPBA on the C-terminus (Scheme 1; Supplementary Material). Synthesis of these insulin analogs followed previously published methods with slight modifications (Liu et al., 2013; 2014; Qiu et al., 2019). The A chain and B chain were first synthesized separately followed by chain combinations. To construct three disulfide bonds in a controlled manner, four Cys of the A chain were introduced with four orthogonal protecting groups used as previously reported (Liu et al., 2013; 2014; Qiu et al., 2019). After the fully protected A chain was synthesized on resin, A6 Cys (S^*t*^Bu) was deprotected by 2-mercaptoethanol and then activated with 2,2′-dithiobis (5-nitropyridine) (DTNP) followed by A11 Cys (Mmt) deprotection under 1% trifluoroacetic acid (TFA) to yield a thiol group. The intramolecular disulfide bond between A6 and A11 was spontaneously formed through a disulfide substitution reaction. The A chain was finally cleaved from the resin to give the A6-A11 disulfide, A7 Cys (Acm), and A20 free Cys A chain **1**. To avoid degradation of FPBA under harsh peptide synthesis reaction conditions, FPBA was introduced at late stage after the whole B chain was synthesized. Fmoc-Lys (Dde)-OH was used for FPBA conjugation with Boc-Phe-OH used for PheB1. After the fully protected B chain was synthesized on resin, all Lys (Dde) residues were deprotected using 5% hydrazine and then coupled with 4-carboxy-3-fluorophenylboronic acid on the ε-amino group to yield the K* residues (Qiu et al., 2019). The B chain was cleaved under a standard cleavage condition in the presence of 2,2′-dithiodipyridine (DTDP) to give the B7 Cys (Acm) and B19 Cys (SPy) B chain **2**. The A and B chain were combined through a disulfide substitution reaction between A20 and B19. The last disulfide bond between A7 and B7 was formed by treatment of iodine. The Thr-Ser isoacyl linkage (to increase solubility of A chain) was finally transformed to an amide bond through an *O*-to-*N* acyl shift under a basic condition to give insulin analogs **4**. We then measured solubilities of insulin analogs at 0–400 mg dl^−1^ glucose solutions to determine their glucose-dependent solubilities (Figure 2A). In the absence of glucose (baseline solubility), insulin analog **4b** (with 3 FPBAs on the B chain) was less soluble compared to the 2FPBA analog **4a** due to the hydrophobicity of FPBA (Figure 2A). However, both analogs demonstrated similar glucose responsive profiles (Figure 2B). Both **4a** and **4b** had comparable EC_50_ with human insulin in activating insulin receptor signaling using pAKT as an indicator (Figure 2C).

**SCHEME 2:**
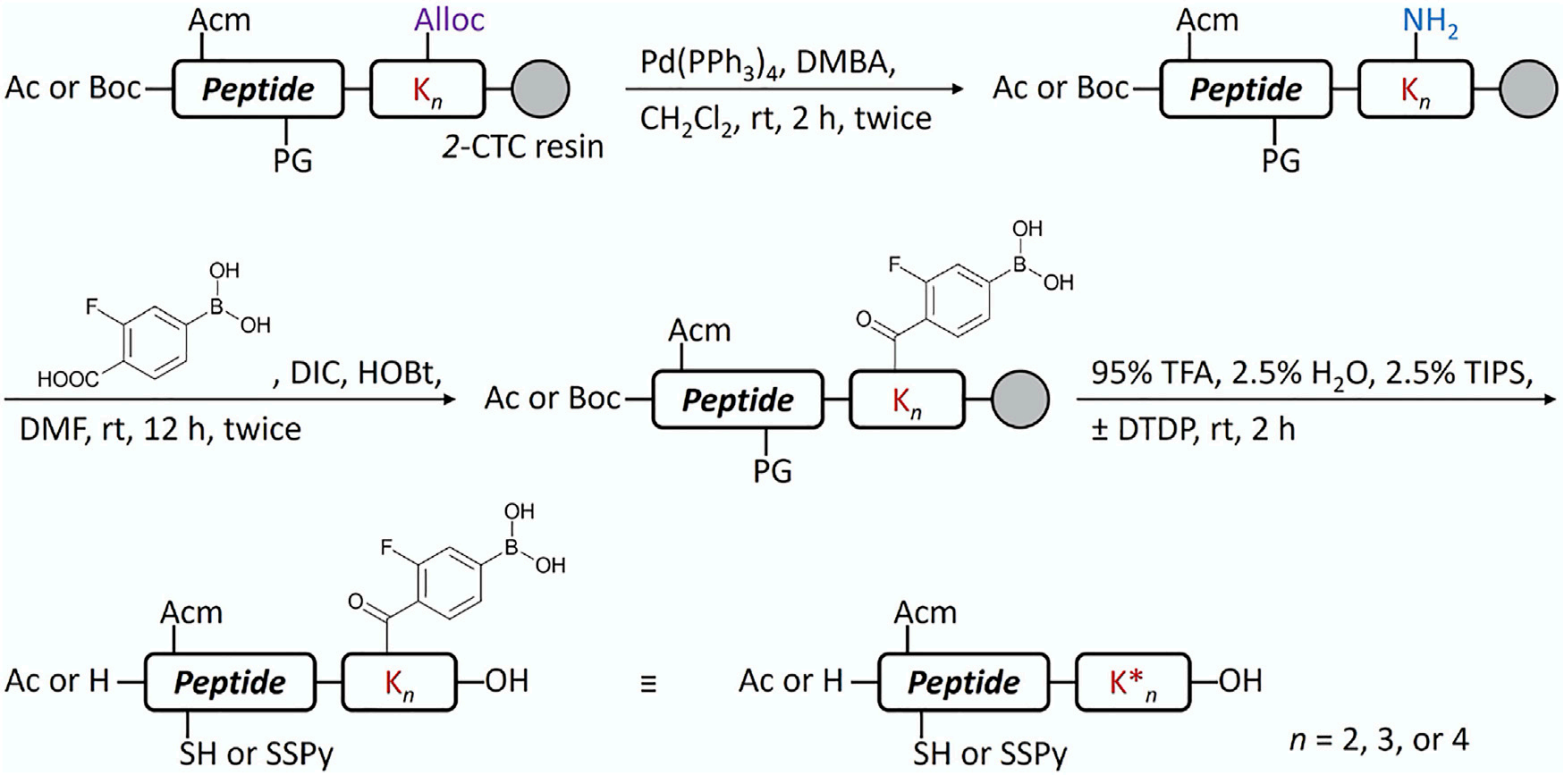
Conjugation of FPBA to peptides by Alloc strategy.

**SCHEME 3:**
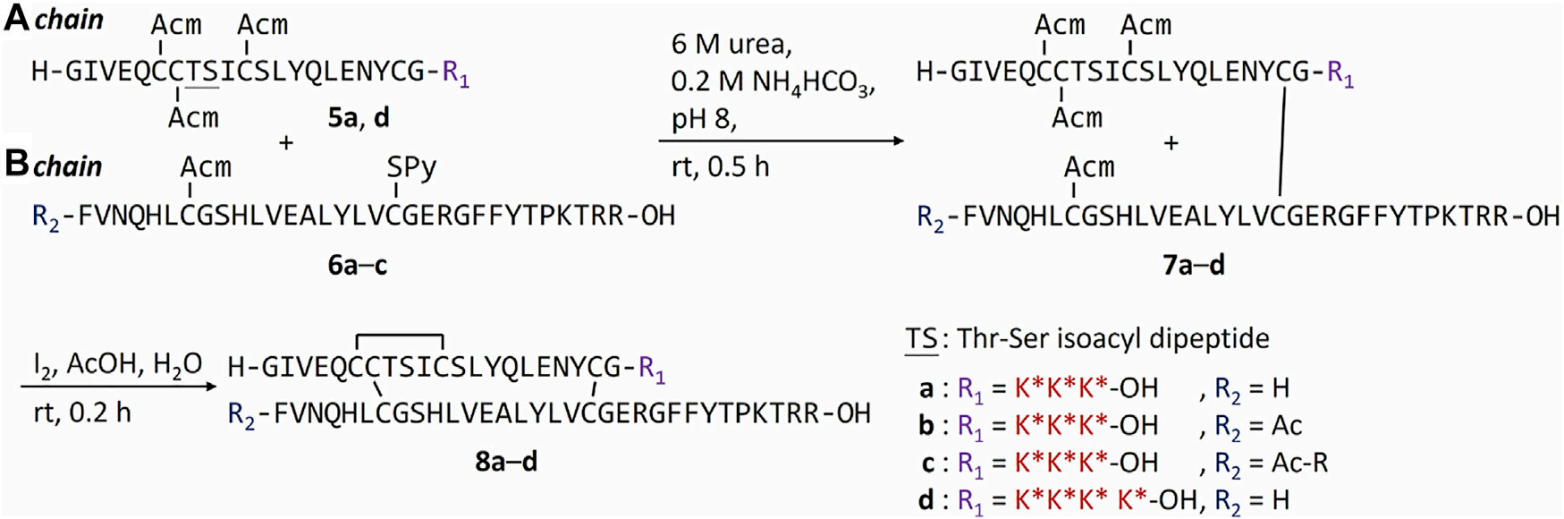
Synthesis of A chain FPBA-conjugated insulin analogs.

**FIGURE 3:**
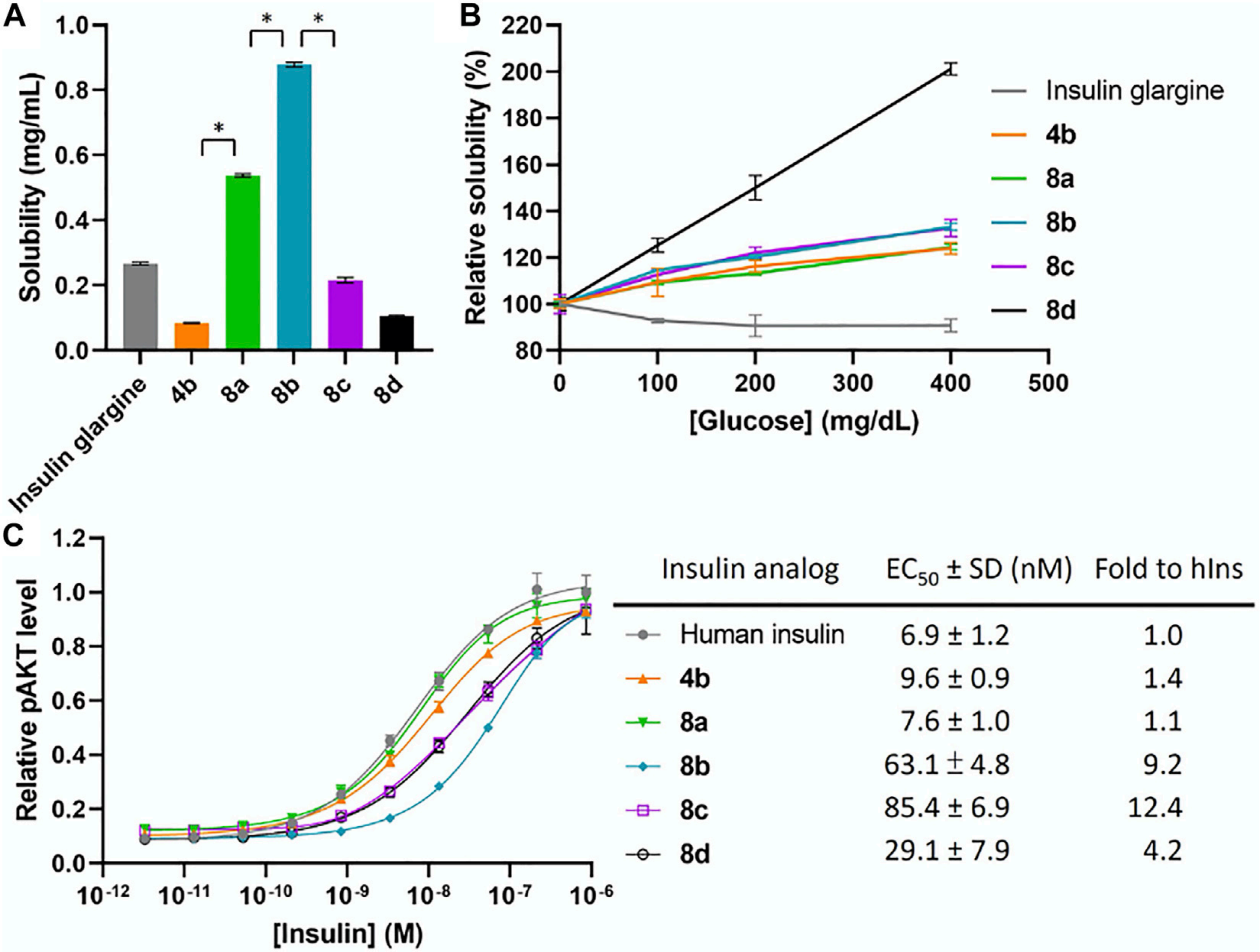
**(A)** Solubility of insulin analogs in pH 7.4 PBS. Data are expressed as mean ± SD (*n* = 4 per group). The statistical comparison of the two groups was evaluated by the unpaired two-tailed Student’s t-test. *p < 0.001. **(B)** Solubility of insulin analogs in pH 7.4 PBS with different glucose concentrations. Data are expressed as mean ± SD (*n* = 4 per group). **(C)** *In vitro* activity of insulin analogs by using activated pAKT levels as measurements. Data are expressed as mean ± SD (*n* = 4 per group). EC_50_ was calculated by Prism 9 (GraphPad Software, CA, United States) with nonlinear regression curve fitting of dose-response asymmetric equation.

Another potential approach to circumvent the solubility issue of B chain intermediates is using sortase A (SrtA)-mediated ligation to semi-synthesize insulin analogs with B chain C-terminal modifications (Disotuar et al., 2021). However, SrtA ligation needs a specific recognition sequence around the ligation site, which becomes a functionless region between insulin and the ligated peptide. Instead, we turn to the C-terminal A chain as an alternative strategy to circumvent the solubility issue. Traditionally, very few publications reported modifications on the C-terminal A chain of insulin (Edgerton et al., 2014; 2019). Our recent work on characterizing venom insulins from fish-hunting cone snails suggests that modifications can be introduced to this region without blocking its ability to bind insulin receptor (Ahorukomeye et al., 2019; Xiong et al., 2020; 2021). In the original synthesis (Scheme 1), 1-(4,4-dimethyl-2,6-dioxocyclohex-1-ylidene)ethyl (Dde) was the key protecting group to allow selective coupling with FPBA. However, Thr-Ser isoacyl linkage was crucial to provide the overall solubility of the A chain but it cannot survive the condition for Dde removal (Liu et al., 2014). To address this challenge, we turn to another orthogonal protecting group, allyloxycarbonyl (Alloc), which uses a mild Pd-mediated deprotection condition (Scheme 2). In addition, to avoid the interference between the Pd catalyst and the A6-A11 intramolecular disulfide on the A chain, both Cys were alternatively protected as Cys (Acm) and the disulfide bond was formed later together with the iodine-mediated disulfide formation of A7-B7 (Liu et al., 2013; Xiong et al., 2020). A total of 4 analogs with FPBA groups on the A chain were synthesized and further evaluated (Scheme 3; Supplementary Material). We found that the solubility of **8a** was improved about 6-fold without a significant change of EC_50_ and glucose responsiveness comparing to the corresponding B chain three FPBA analog **4b** (Figure 3). Acetylation on the B chain N-terminus of **8a** (**8b**) can block the N-terminal amino group, which reduces pI and increases solubility at pH 7.4 (Figure 3A) but EC_50_ had an about 8-fold decrease (Figure 3C). Restoring pI of **8b** by adding an extra Arg on the N-terminal of the B chain (**8c**) decreases the solubility, as expected (Figure 3A). Over-modifications of **8c** had deleterious effects on both solubility and EC_50_ to the insulin receptor (Figure 3C). All three analogs (**8a**, **8b**, and **8c**) had similar glucose-dependent solubility profiles, consistent with the same number of FPBA groups on each analog (Figure 3B).

Due to the increase of overall solubility by relocating FPBAs to the A chain, we were able to synthesize the four FPBA insulin analog **8d**. The baseline solubility of **8d** is comparable to **4a** and **4b**, which have two and three FPBA on B chain respectively (Figure 3A). EC_50_ of **8d** was also only reduced by about 4-fold compared to human insulin (Figure 3C). On the other hand, the additional FPBA brought a jump of glucose-dependent solubility to 100% increase between 0 and 400 mg dl^−1^, in which the corresponding three FPBA analog **8a** just had around 20% (Figure 3B). This result suggests that increasing the number of FPBA groups led to larger glucose-dependent solubility enhancements.

In summary, we explored the conjugation of FPBA groups on insulin C-terminal A chain through a new Alloc-mediated conjugation strategy. We further discovered that by relocating the hydrophobic FPBA residues to the A chain, the baseline solubility of insulin is increased without potency reduction. This allowed further demonstration that increasing FPBA groups led to increasing glucose-dependent solubility. With these exciting findings, further *in vivo* properties are being investigated.

## 3 Materials and Methods

### 3.1 General Information

All Fmoc amino acids, reagents, and solvents were used without purification. Fmoc amino acids and 1-[bis(dimethylamino)methylene]-1*H*-1,2,3-triazolo [4,5-*b*]pyridinium 3-oxid hexafluorophosphate (HATU) were purchase from AAPPTec, Bachem, Chem-Impex, ChemPep, and PurePep. The Rink amide ChemMatrix resin (catalog number: 7-600-1310) was purchased from Biotage. The 2-chlorotrityl chloride (2-CTC) resin (catalog number: 150301) were purchased from ChemPep. *N*,*N*-dimethylformamide (DMF), CH_2_Cl_2_, MeCN, MeOH, Et_2_O, AcOH, Ac_2_O, *N*,*N*-diisopropylethylamine (DIPEA), trifluoroacetic acid (TFA), NH_4_HCl, NH_4_HCO_3_, urea, NaOH, 80% hydrazine hydrate, 2-mercaptoethanol were purchased from Fisher Scientific. Piperidine, triisopropylsilane (TIPS), hydroxybenzotriazole (HOBt), *N*,*N*′-diisopropylcarbodiimide (DIC), 2,2′-dithiodipyridine (DTDP), 2,2′-dithiobis (5-nitropyridine) (DTNP), I_2_, Pd(PPh_3_)_4_, 1,3-dimethylbarbituric acid (DMBA), glucose, sodium ascorbate were purchased from Sigma Aldrich. 4-carboxy-3-fluorophenylboronic acid was purchased from Alfa Aesar. Agilent 6120 Quadrupole LC‒MS system was used to acquire the LC chromatograms and mass spectra of samples with Luna^®^ 5 μm C18 100 Å (50 × 2 mm) column (Phenomenex, CA, United States) at 0.4 ml/min with 5% of a H_2_O/MeCN + 0.1% TFA solution for 1 min followed by a linear gradient from 5% to 95% of a H_2_O/MeCN + 0.1% TFA solution over 5 min.

### 3.2 Automated Fmoc/^*t*^Bu SPPS

Peptides were synthesized *via* Fmoc/^*t*^Bu solid-phase peptide synthesis on Syro I (MultiSynTech GmbH, Germany) in a 10 ml reactor vial with a 0.1 mmol total loading capacity of resin. The first C-terminal amino acid of carboxylic acid C-terminus was coupled manually to the 2-CTC resin: The Fmoc-amino acid (0.1 mmol) and DIPEA (87.1 μl, 0.5 mmol) were dissolved in a solution of DMF and CH_2_Cl_2_ (1:1, 2.5 ml). This solution was added to the 2-CTC resin (250 mg), which was washed with DMF 3 times and then CH_2_Cl_2_ 3 times before the reaction. The reaction mixture was mixed on a rotator for 2 h at room temperature. The resin was washed with DMF 3 times and CH_2_Cl_2_ 3 times and then capped with a solution of CH_2_Cl_2_, MeOH, and DIPEA (17:2:1, 5.0 ml) for 10 s 4 times. The resin was finally washed with CH_2_Cl_2_ 3 times and then DMF 3 times. The first C-terminal amino acid of an amide C-terminus was coupled with Rink Amide resin with the same reaction condition as the typical amino acid coupling as follows. Fmoc was deprotected with 20% piperidine in DMF for 10 min twice at room temperature. Fmoc amino acids were coupled onto resin with a solution of Fmoc-amino acid (0.5 mmol), HATU (0.5 mmol), and DIPEA (1.0 mmol) in DMF (2.5 ml) for 10 min at 50°C (Cys and His) or at 70°C (others). The resin was washed with DMF 3 times between Fmoc deprotection and amino acid coupling.

### 3.3 Peptide Cleavage From Resin

A peptide was cleaved from resins (0.1 mmol) with a solution of TFA, H_2_O, and TIPS (38:1:1, 8 ml) at room temperature for 2 h. Additional DTDP (220 mg, 10 mmol) was added in the cleavage solution to re-protect the thiol group of Cys with thiopyridine (SPy) if required. Peptides were precipitated from the cleavage solutions by adding to cold Et_2_O (80 ml). After centrifuge under 3,000 ×g for 3 min, the supernatant was discarded, and the pellet of peptide was resuspended with Et_2_O (40 ml) and again centrifuged to pellet 2 more times. Then crude material was dried under reduced pressure.

### 3.4 On Resin *N*-Terminal Acetylation

The resin (0.1 mmol) was swelled with DMF for 10 min and then DMF was removed by suction. A solution of Ac_2_O (94.5 μlL, 1.0 mmol) and DIPEA (174 μl, 1.0 mmol) in DMF (4.0 ml) was added to the resin. The reaction mixture was gently agitated at room temperature for 1 h. The solution was removed by suction and the resin was washed with DMF 3 times.

### 3.5 On Resin Lys(Dde) Deprotection

The resin (0.1 mmol) was swelled with DMF for 10 min and then DMF was removed by suction. A 50% hydrazine solution in DMF (4 ml, prepared from 80% hydrazine hydrate) was added to the resin. The reaction mixture was gently agitated at room temperature for 30 min. The solution was removed by suction and the resin was washed with DMF 3 times.

### 3.6 On Resin Lys(Alloc) Deprotection

The resin (0.1 mmol) was swelled with CH_2_Cl_2_ for 10 min and then CH_2_Cl_2_ was removed by suction. A solution of Pd(PPh_3_)_4_ (11.6 mg per Alloc, 0.01 mmol per Alloc) and DMBA (31.2 mg per Alloc, 0.2 mmol per Alloc) in CH_2_Cl_2_ (4 ml) was added to the resin. The reaction mixture was gently agitated at room temperature for 2 h. The solution was removed by suction and the resin was washed with CH_2_Cl_2_ 3 times. The reaction was monitored by LC‒MS with microcleavage of the resin. The above treatment was repeated until all Alloc were removed.

### 3.7 On Resin Conjugation of FPBA to Lys

The resin (0.1 mmol) was swelled with DMF for 10 min and then DMF was removed by suction. A solution of 4-carboxy-3-fluorophenylboronic acid (55.2 mg per Lys, 0.3 mmol per Lys), DIC (47.0 μl per Lys, 0.3 mmol per Lys), and HOBt (40.5 mg per Lys, 0.3 mmol per Lys) in DMF (4 ml) was gently agitated at room temperature for 10 min and then added to the resin. The reaction mixture was gently agitated at room temperature for 12 h. The solution was removed by suction and the resin was washed with DMF 3 times. The reaction was monitored by LC‒MS with microcleavage of the resin. The above treatment was repeated until all Lys were conjugated with FPBA.

### 3.8 General Synthetic Procedure of 1

Peptide **1** was synthesized by following the general procedure of automated Fmoc/^*t*^Bu SPPS. Isoacyl-dipeptide Boc-Ser [Fmoc-Thr (^*t*^Bu)]-OH was used to create the isoacyl linkage between ThrA8 and SerA9. After the entire sequence was completed on resin, on resin formation of A6-A11 disulfide bond was carried out by following the reported procedure (Liu et al., 2014). The resin was treated with 25% 2-mercaptoethanol in DMF (*v*/*v*, 6 ml) at room temperature for 1.5 h with gentle agitation. This step was repeated once. The resulting resin was washed with DMF 3 times and CH_2_Cl_2_ 3 times. A solution of DTNP (310 mg, 1 mmol) in CH_2_Cl_2_ (6 ml) was added to the resin. The reaction mixture was gently agitated at room temperature for 1.0 h. The resin was washed with DMF 3 times and CH_2_Cl_2_ 3 times. The resin was and treated with a solution of 1% TFA and 5% TIPS in CH_2_Cl_2_ (6 ml) for 2 min with 5 repeats. The resin was washed with DMF 3 times and CH_2_Cl_2_ 3 times and gently agitated in CH_2_Cl_2_ (6 ml) at room temperature for 1 h. The resin was washed with CH_2_Cl_2_ 3 times. The final peptide was cleaved by following the general procedure of peptide cleavage from resin. The crude was purified on Luna^®^ 5 μm C18 100 Å (250 × 21 mm) column (Phenomenex, CA, United States) at 5 ml/min with a linear gradient from 30% to 50% of a H_2_O/MeCN + 0.1% TFA solution over 30 min on an Agilent 1260 HPLC system detected at 220, 240, 260, and 280 nm. The fractions containing **1** were flash frozen under liquid N_2_ and then lyophilized to give **1** as a white powder.

### 3.9 General Synthetic Procedure of 2

Peptide **2** was synthesized by following the general procedure of automated Fmoc/^*t*^Bu SPPS. Fmoc-Lys (Dde)-OH was used for FPBA conjugation and Boc-Phe-OH was used for PheB1. After the entire sequence was completed on resin, Lys (Dde) was deprotected by following the general procedure of on resin Lys (Dde) deprotection and then FPBA was introduced by following the general procedure of on resin conjugation of FPBA to Lys. The final peptide was cleaved by following the general procedure of peptide cleavage from resin. The crude was purified on Luna^®^ 5 μm C18 100 Å (250 × 21 mm) column (Phenomenex, CA, United States) at 5 ml/min with a linear gradient from 20% to 60% of a H_2_O/MeCN + 0.1% TFA solution over 40 min on an Agilent 1260 HPLC system at 220, 240, 260, and 280 nm. The fractions containing **2** were flash frozen under liquid N_2_ and then lyophilized to give **2** as a white powder.

### 3.10 General Synthetic Procedure of 3

The lyophilized A chain powder **1** (1.0 μmol) and the B chain powder **2** (1.0 μmol) were each dissolved in a solution of 6 M urea and 0.2 M NH_4_Cl (pH 5, 0.25 ml). The solutions of **1** and **2** were mixed with equal volumes (0.25 ml). The reaction mixture was gently mixed and left at room temperature for 4 h. The resulting solution was purified on Jupiter^®^ 5 μm C18 300 Å (250 × 10 mm) column (Phenomenex, CA, United States) at 3 ml/min with a linear gradient from 10% to 60% of a H_2_O/MeCN + 0.1% TFA solution over 50 min on an Agilent 1260 HPLC system detected at 220, 240, 260, and 280 nm. The fractions containing **3** were flash frozen under liquid N_2_ and then lyophilized to give **3** as a white powder.

### 3.11 General Synthetic Procedure of 4

The lyophilized powder **3** (1.0 μmol) was dissolved in a solution of 20% AcOH in H_2_O (*v*/*v*, 0.33 ml). A freshly prepared solution of I_2_ (2.54 mg, 10 μmol) in AcOH (0.5 ml) was added into the solution of **3** at room temperature. The reaction mixture was gently agitated at room temperature for 10 min. The reaction was monitored by LC‒MS to apply the additional amount of I_2_ portionwise if required. After the reaction was completed, a solution of 1 M sodium ascorbate (0.3 ml) was added to the reaction mixture followed by gentle agitation until color of the solution turned to pale yellow. After added with a solution of 0.1% TFA in H_2_O (1.5 ml), the reaction mixture was purified on Jupiter^®^ 5 μm C18 300 Å (250 × 10 mm) column (Phenomenex, CA, United States) at 3 ml/min with a linear gradient from 10% to 60% of a H_2_O/MeCN + 0.1% TFA solution over 50 min on an Agilent 1260 HPLC system detected at 220, 240, 260, and 280 nm. The fractions with resulting product were flash frozen under liquid N_2_ and then lyophilized to give a white powder. The white powder was dissolved in 0.2 M NH_4_HCO_3_ (pH 8). The reaction mixture was gently agitated at room temperature for 30 min and then purified on Jupiter^®^ 5 μm C18 300 Å (250 × 10 mm) column (Phenomenex, CA, United States) at 3 ml/min with a linear gradient from 10% to 60% of a H_2_O/MeCN + 0.1% TFA solution over 50 min on an Agilent 1260 HPLC system detected at 220, 240, 260, and 280 nm. The fractions containing **4** were flash frozen under liquid N_2_ and then lyophilized to give **4** as a white powder.

### 3.12 General Synthetic Procedure of 5

Peptide **5** was synthesized by following the general procedure of automated Fmoc/^*t*^Bu SPPS. Isoacyl-dipeptide Boc-Ser[Fmoc-Thr (^*t*^Bu)]-OH was used to create the isoacyl linkage between ThrA8 and SerA9. Fmoc-Lys(Alloc)-OH was used for FPBA conjugation and Boc-Gly-OH was used for GlyA1. After the entire sequence was completed on resin, Lys(Alloc) was deprotected by following the general procedure of on resin Lys(Alloc) deprotection and then FPBA was introduced by following the general procedure of on resin conjugation of FPBA to Lys. The final peptide was cleaved by following the general procedure of peptide cleavage from resin. The crude was purified on Luna^®^ 5 μm C18 100 Å (250 × 21 mm) column (Phenomenex, CA, United States) at 5 ml/min with a linear gradient from 30% to 50% of a H_2_O/MeCN + 0.1% TFA solution over 30 min on an Agilent 1260 HPLC system detected at 220, 240, 260, and 280 nm. The fractions containing **5** were flash frozen under liquid N_2_ and then lyophilized to give **5** as a white powder.

### 3.13 General Synthetic Procedure of 6

Peptide **6** was synthesized by following the general procedure of automated Fmoc/^*t*^Bu SPPS. If required, *N*-terminal acetylation was carried out by following the general procedure of on resin *N*-terminal acetylation. The final peptide was cleaved by following the general procedure of peptide cleavage from resin. The crude was purified on Luna^®^ 5 μm C18 100 Å (250 × 21 mm) column (Phenomenex, CA, United States) at 5 ml/min with a linear gradient from 20% to 60% of a H_2_O/MeCN + 0.1% TFA solution over 40 min on an Agilent 1260 HPLC system at 220, 240, 260, and 280 nm. The fractions containing **6** were flash frozen under liquid N_2_ and then lyophilized to give **6** as a white powder.

### 3.14 General Synthetic Procedure of 7

The lyophilized A chain powder **5** (1.0 μmol) and the B chain powder **6** (1.0 μmol) were each dissolved in a solution of 6 M urea and 0.2 M NH_4_Cl (pH 8, 0.25 ml). The solutions of **5** and **6** were mixed with equal volumes (0.25 ml). The reaction mixture was gently mixed and left at room temperature for 30 min. The resulting solution was purified on Jupiter^®^ 5 μm C18 300 Å (250 × 10 mm) column (Phenomenex, CA, United States) at 3 ml/min with a linear gradient from 10% to 60% of a H_2_O/MeCN + 0.1% TFA solution over 50 min on an Agilent 1260 HPLC system detected at 220, 240, 260, and 280 nm. The fractions containing **7** were flash frozen under liquid N_2_ and then lyophilized to give **7** as a white powder.

### 3.15 General Synthetic Procedure of 8

The lyophilized powder **7** (1.0 μmol) was dissolved in a solution of 20% AcOH in H_2_O (*v*/*v*, 0.33 ml). A freshly prepared solution of I_2_ (2.54 mg, 10 μmol) in AcOH (0.5 ml) was added into the solution of **3** at room temperature. The reaction mixture was gently agitated at room temperature for 10 min. The reaction was monitored by LC‒MS to apply the additional amount of I_2_ portion wise if required. After the reaction was completed, a solution of 1 M sodium ascorbate (0.3 ml) was added to the reaction mixture followed by gentle agitation until color of the solution turned to pale yellow. After added with a solution of 0.1% TFA in H_2_O (1.5 ml), the reaction mixture was purified on Jupiter^®^ 5 μm C18 300 Å (250 × 10 mm) column (Phenomenex, CA, United States) at 3 ml/min with a linear gradient from 10% to 60% of a H_2_O/MeCN + 0.1% TFA solution over 50 min on an Agilent 1260 HPLC system detected at 220, 240, 260, and 280 nm. The fractions containing **8** were flash frozen under liquid N_2_ and then lyophilized to give **8** as a white powder.

### 3.16 Solubility Determination

Lyophilized insulin analogs were dissolved with Milli-Q water in 10 mg/mL as a stock solution. An equal ratio of insulin stock solution and 5× phosphate buffered saline (PBS) were mixed. Then, pH of the mixture was adjusted by 100 and 10 mM NaOH solution to 7.4, the glucose solution (2,000 mg/dl) was added to target different final concentrations (0, 100, 200, 400 mg/dl), and the mixture was finally diluted to 1× PBS. The mixtures were gently shaken at room temperature for 2 h and then centrifuged under 20,000 ×g for 10 min. Saturated peptide concentrations of supernatants were determined by the measurements of absorption at 280 nm with NanoDrop One (Thermo Fisher Scientific) and the calculated extinction coefficient at 280 nm (ε_280_). ε_280_ = (number of Trp × 5,500) + (number of Tyr × 1490) + (number of Cystine × 125) + (number of FPBA × 745).

### 3.17 Cell-Based pAKT (Ser473) Assay

The bioactivities of insulin analogs were measured through cell-based pAKT (Ser473) assay. pAKT levels were measured in a human insulin receptor-B overexpressed R^−^ NIH 3T3-like cell line, derived from IGF-1R knockout mice (a generous gift from A. Morrione, Thomas Jefferson University). Cells were cultured in DMEM (Sigma Aldrich) with 10% fetal bovine serum (FBS, Gibco), 100 U/ml penicillin-streptomycin (Thermo Fisher Scientific) and 2 mg/ml puromycin (Thermo Fisher Scientific) at 37 °C under 5% CO_2_. For each assay, 40,000 cells per well and 100 μl per well, were plated in a 96-well plate with culture media containing 1% FBS. 20 h later, the media was removed followed by adding 50 μl of culture media with different concentrations of recombinant human insulin or insulin analogs into each well. After 30-min at 37°C, the insulin solution was removed and the HTRF pAKT Ser473 kit (Cisbio, MA, United States) was used to measure the intracellular level of pAKT Ser473 by following the manufacturer’s protocol. Briefly, cells were first treated with cell lysis buffer (50 μl per well) for 1 h under mild shaking. 16 μl of cell lysate was then added to 4 μl of detecting reagent in a white 384-well plate. After 4-h incubation, the plate was read in a Synergy Neo plate reader (BioTek, VT, United States) or SpectraMax iD5 (Molecular Devices, CA, United States). Each data point was sampled from four replicates. Data were processed according to the manufacturer’s protocol. EC_50_ was calculated by Prism 9 (GraphPad Software, CA, United States) with nonlinear regression curve fitting of dose-response asymmetric equation.

## Data Availability

The raw data supporting the conclusion of this article will be made available by the authors, without undue reservation.
