# Supplementary material for: Synthesis and Characterization of Phenylboronic Acid-Modified Insulin With Glucose-Dependent Solubility
Source: Front Chem. 2022 Mar 16;10:859133. doi: 10.3389/fchem.2022.859133 (PMC8965884; doi:10.3389/fchem.2022.859133)
Supplement: Supplementary file 1 [file DataSheet1.docx]

Supplementary Material

# Supplementary Figures and Tables

Table of contents

| Supplementary Table 1 | MS characterization of insulin analogs |
| --- | --- |
| Supplementary Figure 1 | LC chromatogram and MS spectrum of **1** |
| Supplementary Figure 2 | LC chromatogram and MS spectrum of **2a** |
| Supplementary Figure 3 | LC chromatogram and MS spectrum of **2b** |
| Supplementary Figure 4 | LC chromatogram and MS spectrum of **3a** |
| Supplementary Figure 5 | LC chromatogram and MS spectrum of **3b** |
| Supplementary Figure 6 | LC chromatogram and MS spectrum of **4a** |
| Supplementary Figure 7 | LC chromatogram and MS spectrum of **4b** |
| Supplementary Figure 8 | LC chromatogram and MS spectrum of **5a** |
| Supplementary Figure 9 | LC chromatogram and MS spectrum of **5d** |
| Supplementary Figure 10 | LC chromatogram and MS spectrum of **6a** |
| Supplementary Figure 11 | LC chromatogram and MS spectrum of **6c** |
| Supplementary Figure 12 | LC chromatogram and MS spectrum of **7a** |
| Supplementary Figure 13 | LC chromatogram and MS spectrum of **7c** |
| Supplementary Figure 14 | LC chromatogram and MS spectrum of **7d** |
| Supplementary Figure 15 | LC chromatogram and MS spectrum of **8a** |
| Supplementary Figure 16 | LC chromatogram and MS spectrum of **8b** |
| Supplementary Figure 17 | LC chromatogram and MS spectrum of **8c** |
| Supplementary Figure 18 | LC chromatogram and MS spectrum of **8d** |

## Supplementary Table

Supplementary Table 1. MS characterization of insulin analogs.

|  | **4a** | **4b** | **8a** | **8b** | **8c** | **8d** |
| --- | --- | --- | --- | --- | --- | --- |
| Calculated monoisotopic mass | 6490.0 | 6940.2 | 6941.2 | 6983.2 | 7139.3 | 7235.3 |
| Observed m/z: | | | | | | |
| [M+8H-8H_2_O]^8+^ |  |  |  |  |  | 887.6 |
| [M+8H-7H_2_O]^8+^ |  |  |  |  |  | 889.8 |
| [M+8H-6H_2_O]^8+^ |  |  | 855.5 |  | 880.3 |  |
| [M+8H-5H_2_O]^8+^ |  |  |  |  | 882.5 |  |
| [M+8H-1H_2_O]^8+^ |  |  |  |  |  |  |
| [M+8H]^8+^ |  |  |  |  |  |  |
|  |  |  |  |  |  |  |
| [M+7H-8H_2_O]^7+^ |  |  |  |  |  | 1014.7 |
| [M+7H-7H_2_O]^7+^ |  |  |  |  |  | 1017.2 |
| [M+7H-6H_2_O]^7+^ |  |  | 977.5 | 983.8 | 1005.8 | 1019.5 |
| [M+7H-5H_2_O]^7+^ |  |  | 980.0 | 986.2 | 1008.7 |  |
| [M+7H-4H_2_O]^7+^ |  |  | 983.0 |  | 1011.0 |  |
| [M+7H-1H_2_O]^7+^ |  | 990.3 |  |  |  |  |
| [M+7H]^7+^ | 928.1 | 992.8 |  |  |  |  |
|  |  |  |  |  |  |  |
| [M+6H-8H_2_O]^6+^ |  |  |  |  |  | 1183.4 |
| [M+6H-7H_2_O]^6+^ |  |  |  |  |  | 1186.6 |
| [M+6H-6H_2_O]^6+^ |  |  | 1140.6 | 1147.3 | 1173.6 | 1189.4 |
| [M+6H-5H_2_O]^6+^ |  |  | 1143.6 | 1150.2 | 1176.4 | 1192.8 |
| [M+6H-4H_2_O]^6+^ |  |  | 1146.5 | 1153.4 | 1179.3 |  |
| [M+6H-1H_2_O]^6+^ |  | 1155.1 |  |  |  |  |
| [M+6H]^6+^ | 1082.6 | 1158.2 |  |  |  |  |
|  |  |  |  |  |  |  |
| [M+5H-8H_2_O]^5+^ |  |  |  |  |  | 1420.1 |
| [M+5H-7H_2_O]^5+^ |  |  |  |  |  | 1423.3 |
| [M+5H-6H_2_O]^5+^ |  | 1368.0 | 1368.4 | 1376.6 | 1407.9 | 1427.4 |
| [M+5H-5H_2_O]^5+^ |  | 1371.7 | 1371.9 | 1380.3 | 1411.6 | 1430.7 |
| [M+5H-4H_2_O]^5+^ | 1284.6 | 1375.5 |  | 1383.7 | 1415.1 |  |
| [M+5H-3H_2_O]^5+^ | 1288.2 | 1379.1 |  |  |  |  |
| [M+5H-2H_2_O]^5+^ | 1291.6 |  |  |  |  |  |
| [M+5H-1H_2_O]^5+^ | 1295.3 |  |  |  |  |  |
| [M+5H]^5+^ | 1298.9 |  |  |  |  |  |
|  |  |  |  |  |  |  |
| [M+4H-7H_2_O]^4+^ |  |  |  |  |  | 1778.8 |
| [M+4H-6H_2_O]^4+^ |  |  |  |  |  | 1783.6 |
| [M+4H-5H_2_O]^4+^ |  | 1714.6 | 1714.5 | 1725.0 | 1764.6 | 1787.7 |
| [M+4H-4H_2_O]^4+^ | 1605.4 | 1718.8 | 1719.1 |  | 1768.9 |  |
| [M+4H-3H_2_O]^4+^ | 1610.2 |  |  |  |  |  |

## Supplementary Figures

A


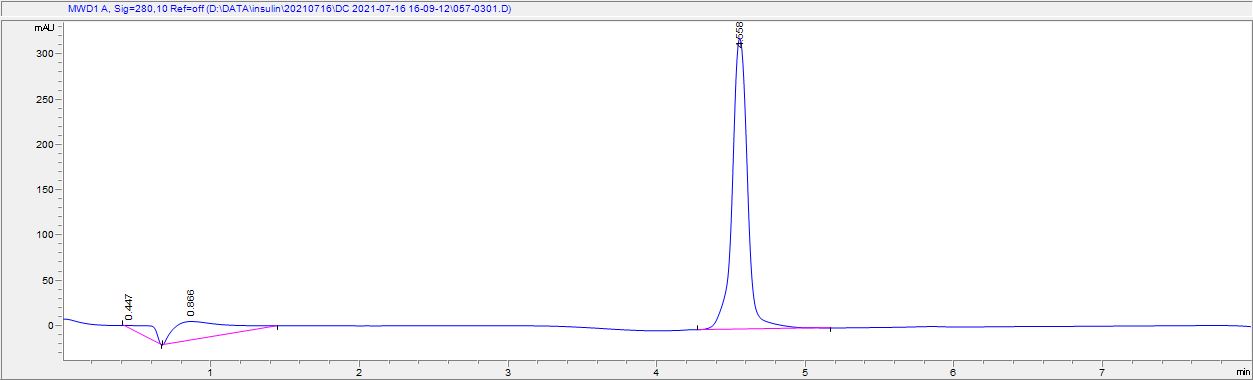


B


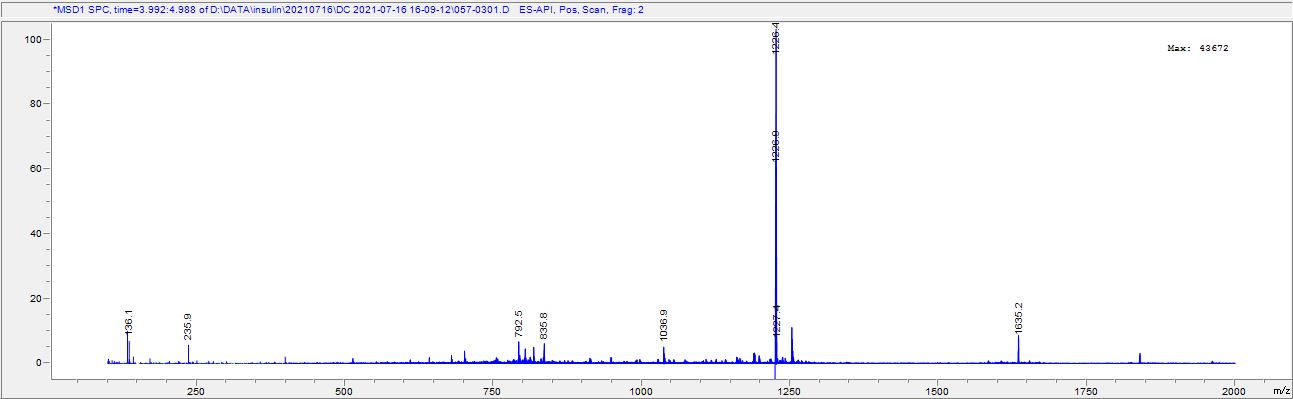


**Supplementary Figure 1.** (A) LC chromatogram (280 nm) and (B) MS spectrum of **1**

A


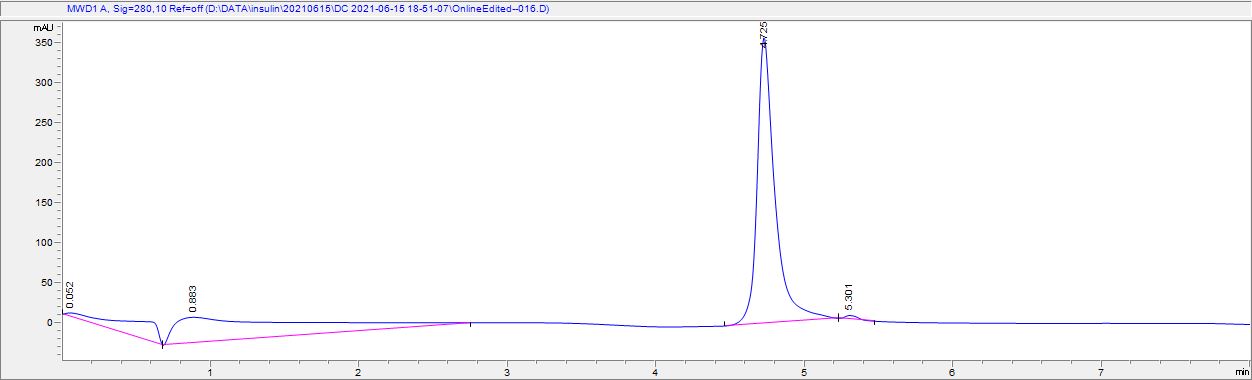


B


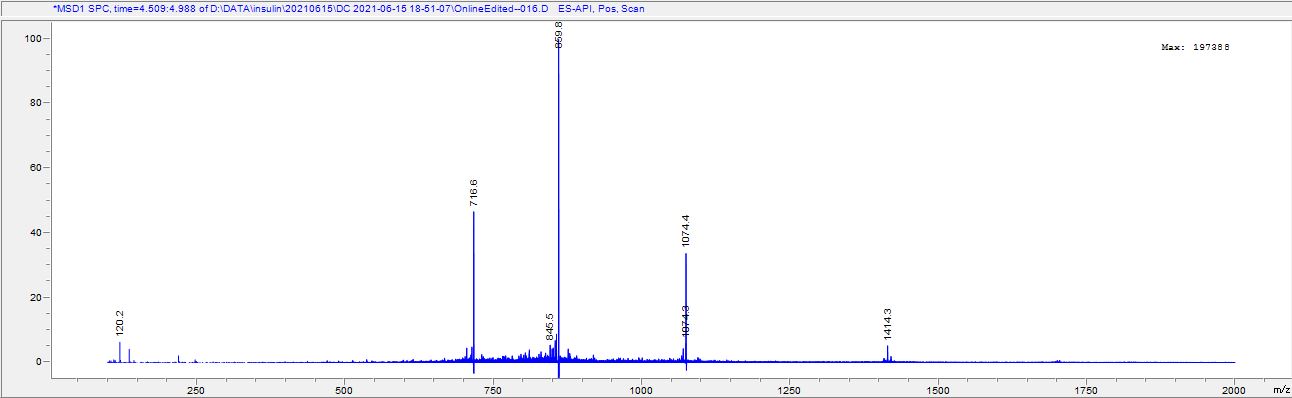


**Supplementary Figure 2.** (A) LC chromatogram (280 nm) and (B) MS spectrum of **2a**

A


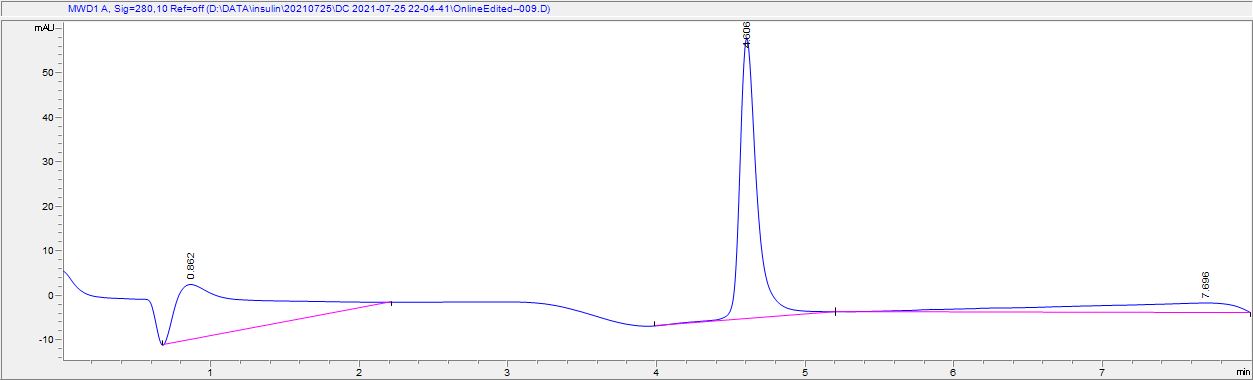


B


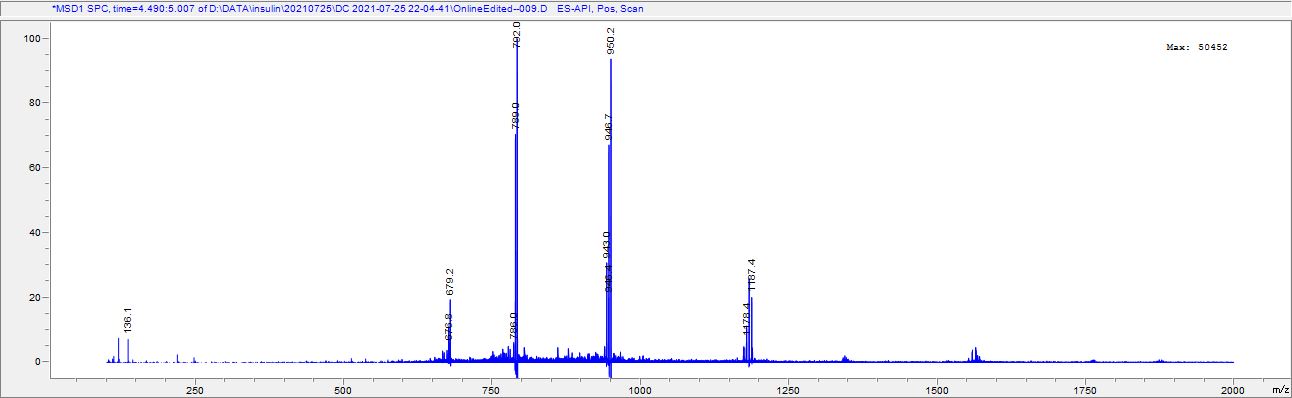


**Supplementary Figure 3.** (A) LC chromatogram (280 nm) and (B) MS spectrum of **2b**

A


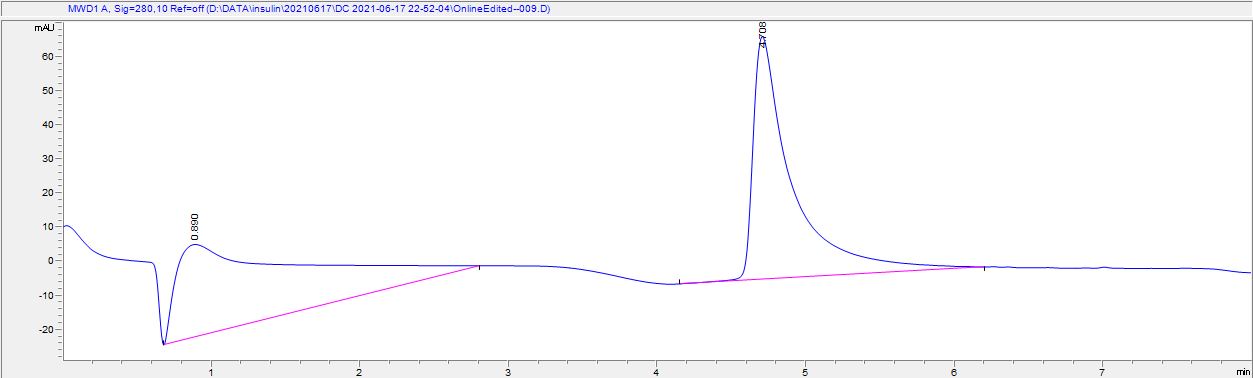


B


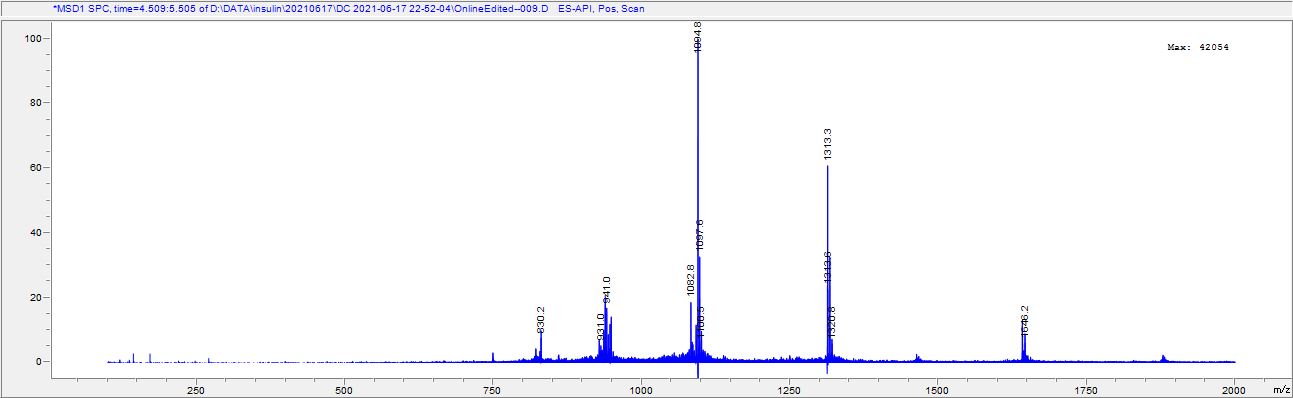


**Supplementary Figure 4.** (A) LC chromatogram (280 nm) and (B) MS spectrum of **3a**

A


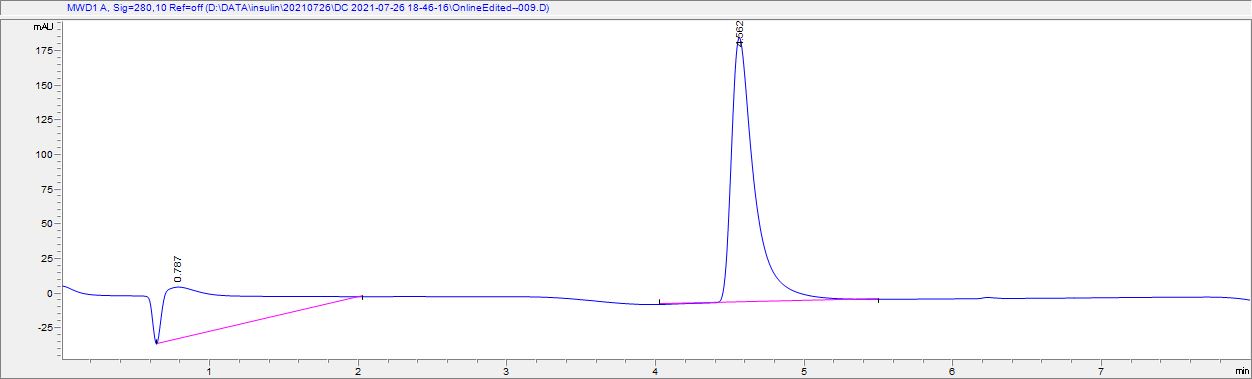


B


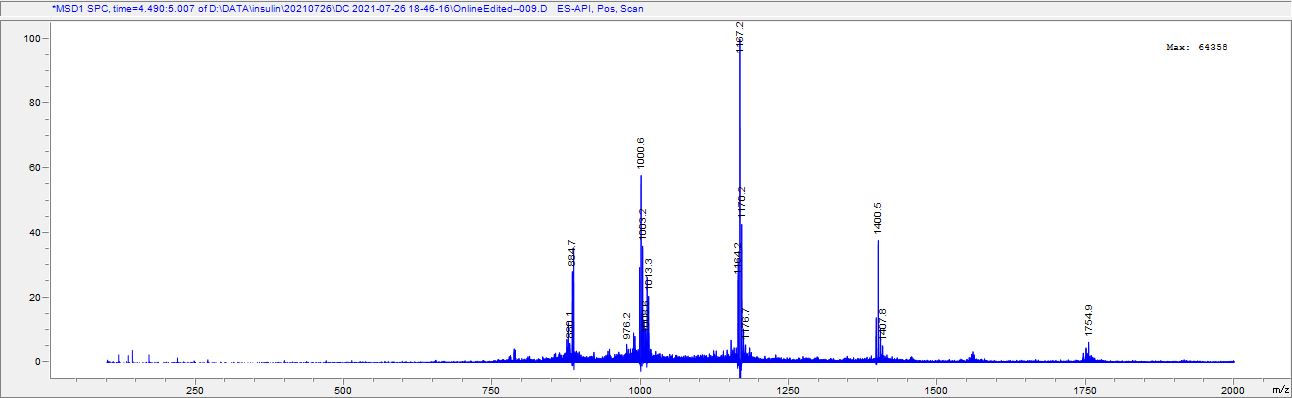


**Supplementary Figure 5.** (A) LC chromatogram (280 nm) and (B) MS spectrum of **3b**

A


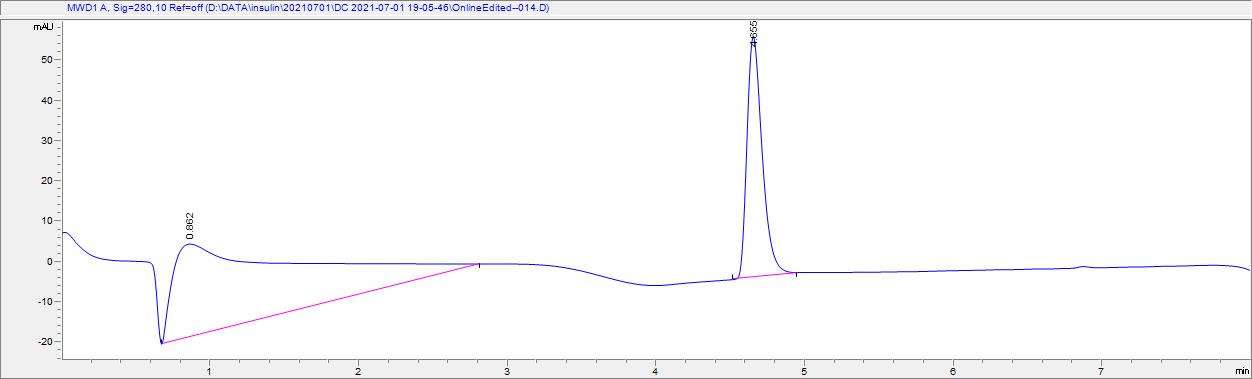


B


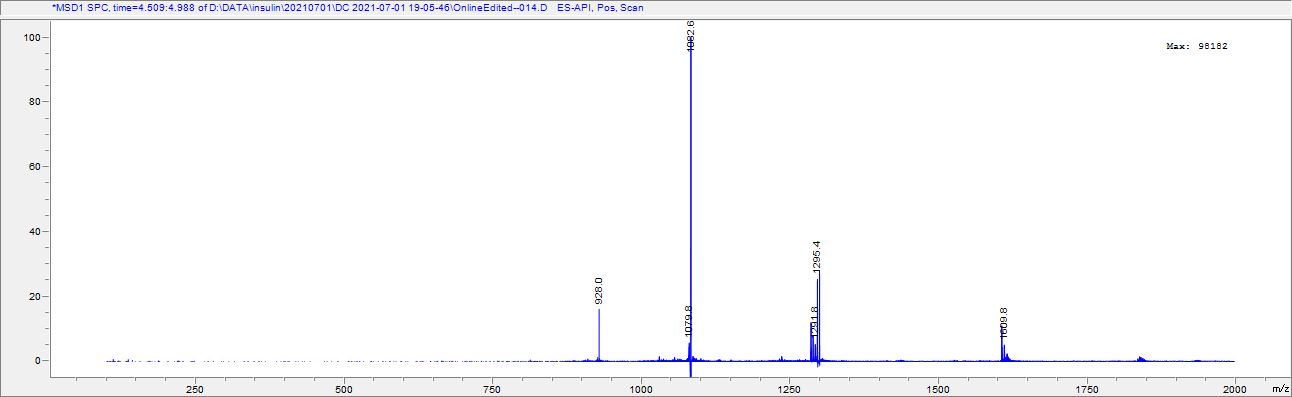


**Supplementary Figure 6.** (A) LC chromatogram (280 nm) and (B) MS spectrum of **4a**

A


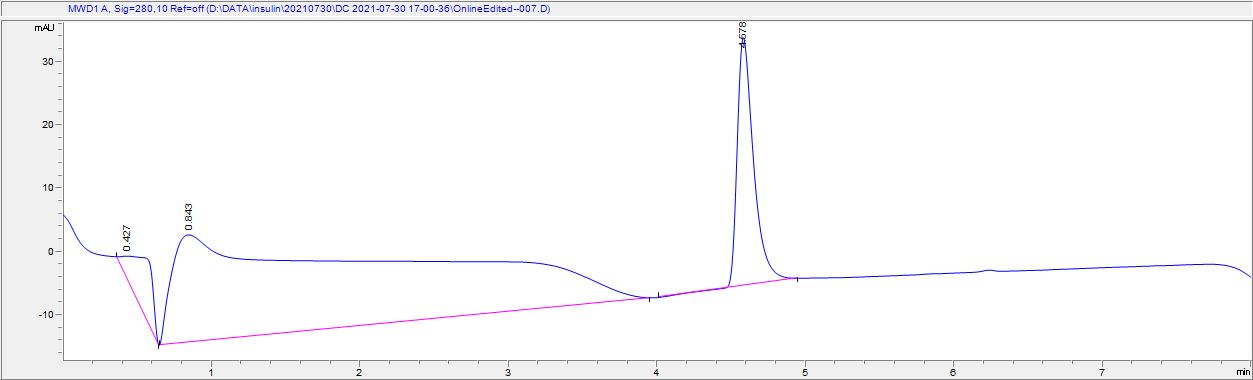


B


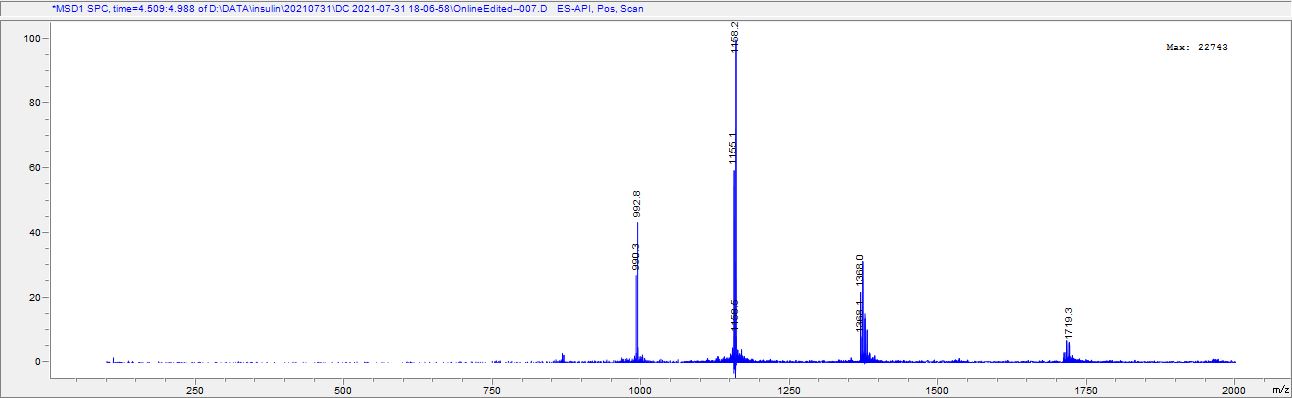


**Supplementary Figure 7.** (A) LC chromatogram (280 nm) and (B) MS spectrum of **4b**

A


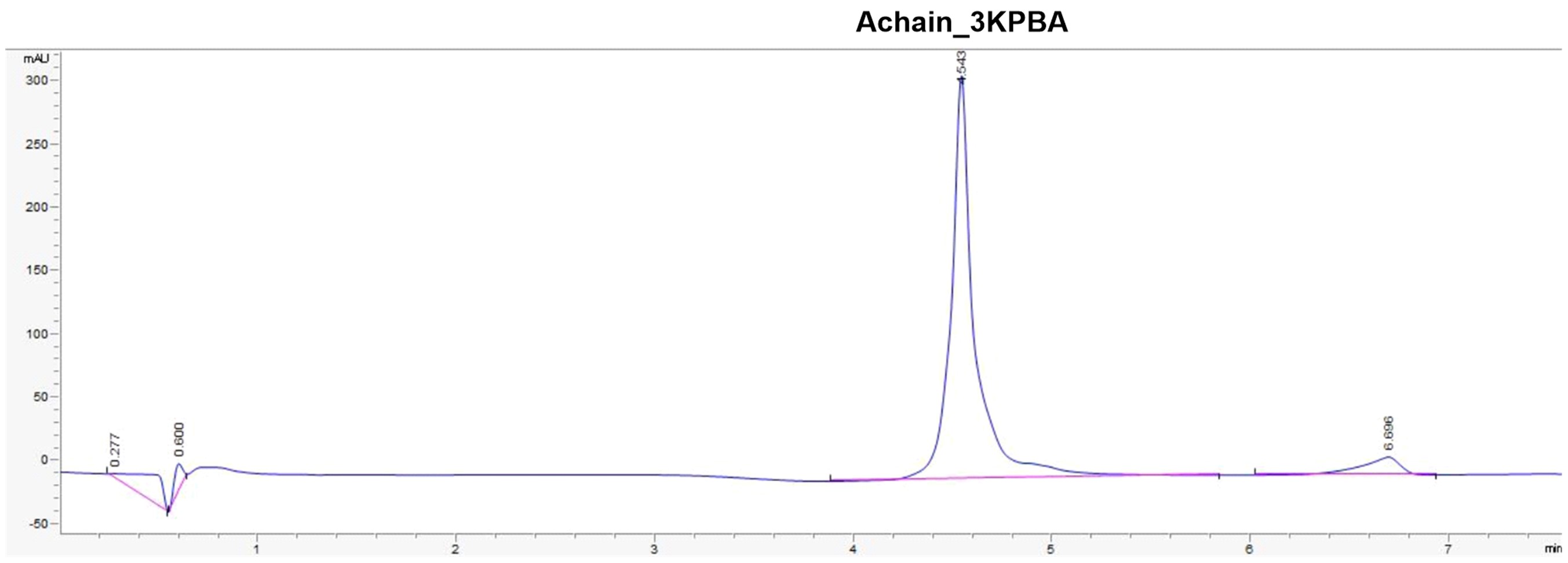


B


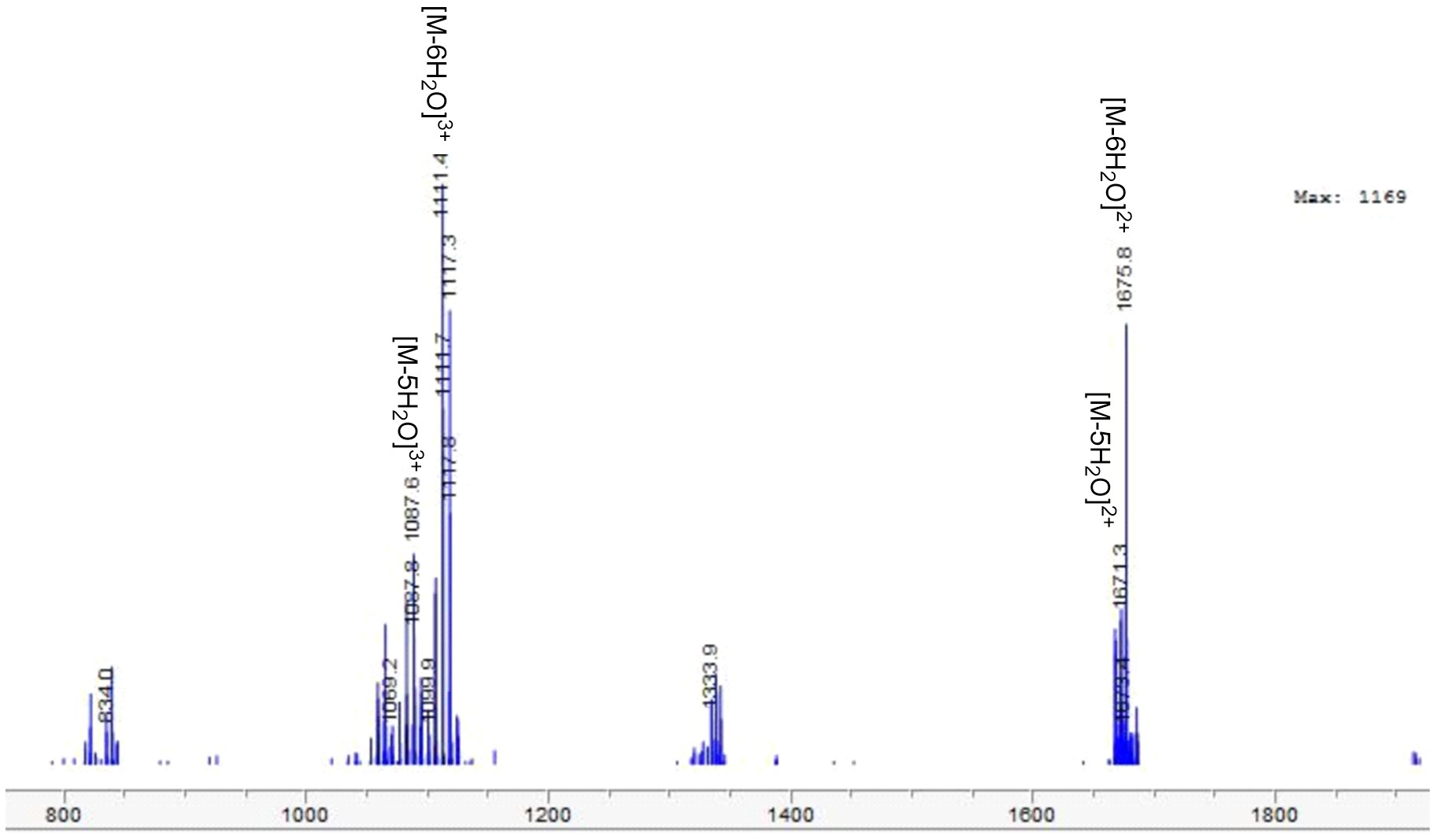


**Supplementary Figure 8.** (A) LC chromatogram (280 nm) and (B) MS spectrum of **5a**

A


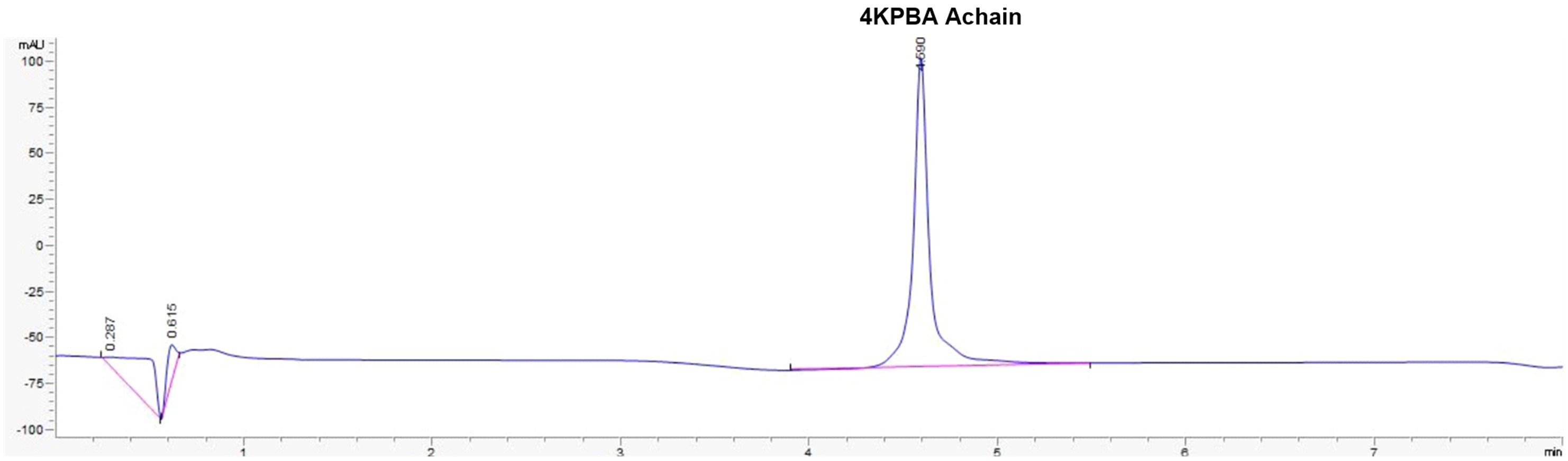


B


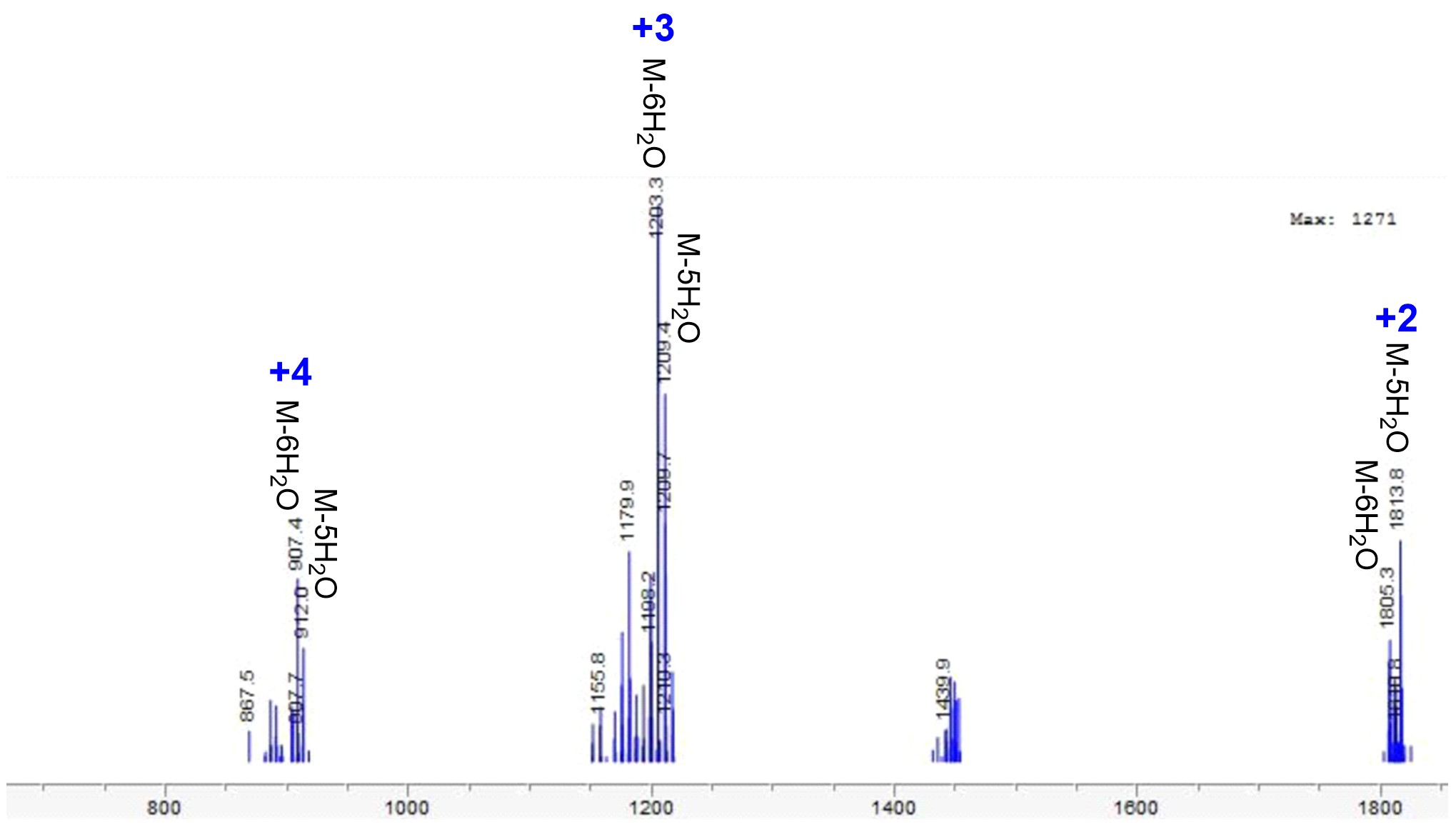


**Supplementary Figure 9.** (A) LC chromatogram (280 nm) and (B) MS spectrum of **5d**

A


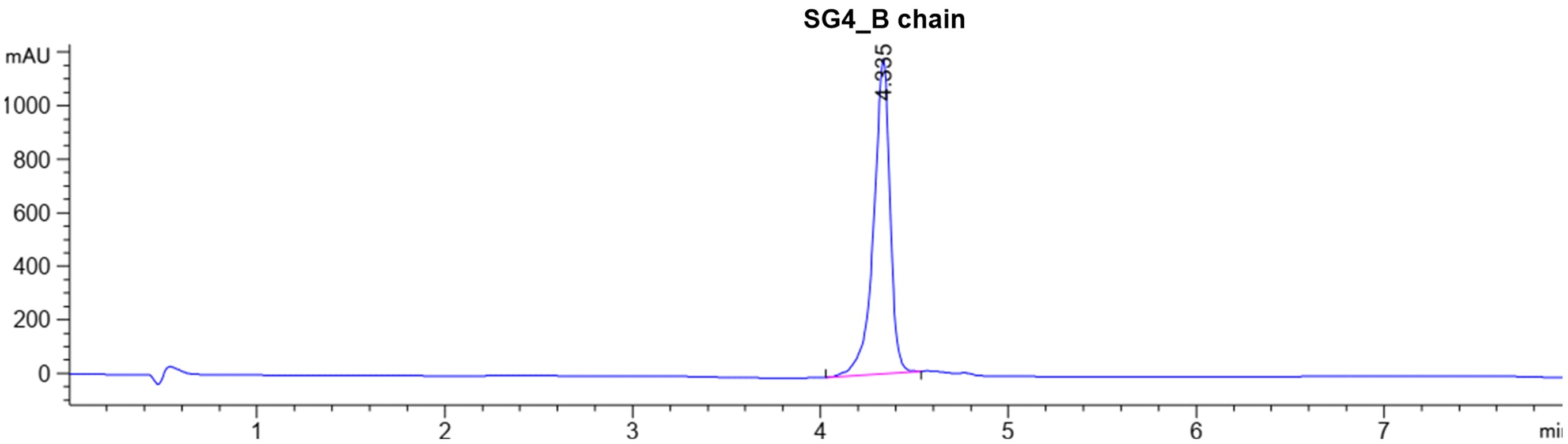


B


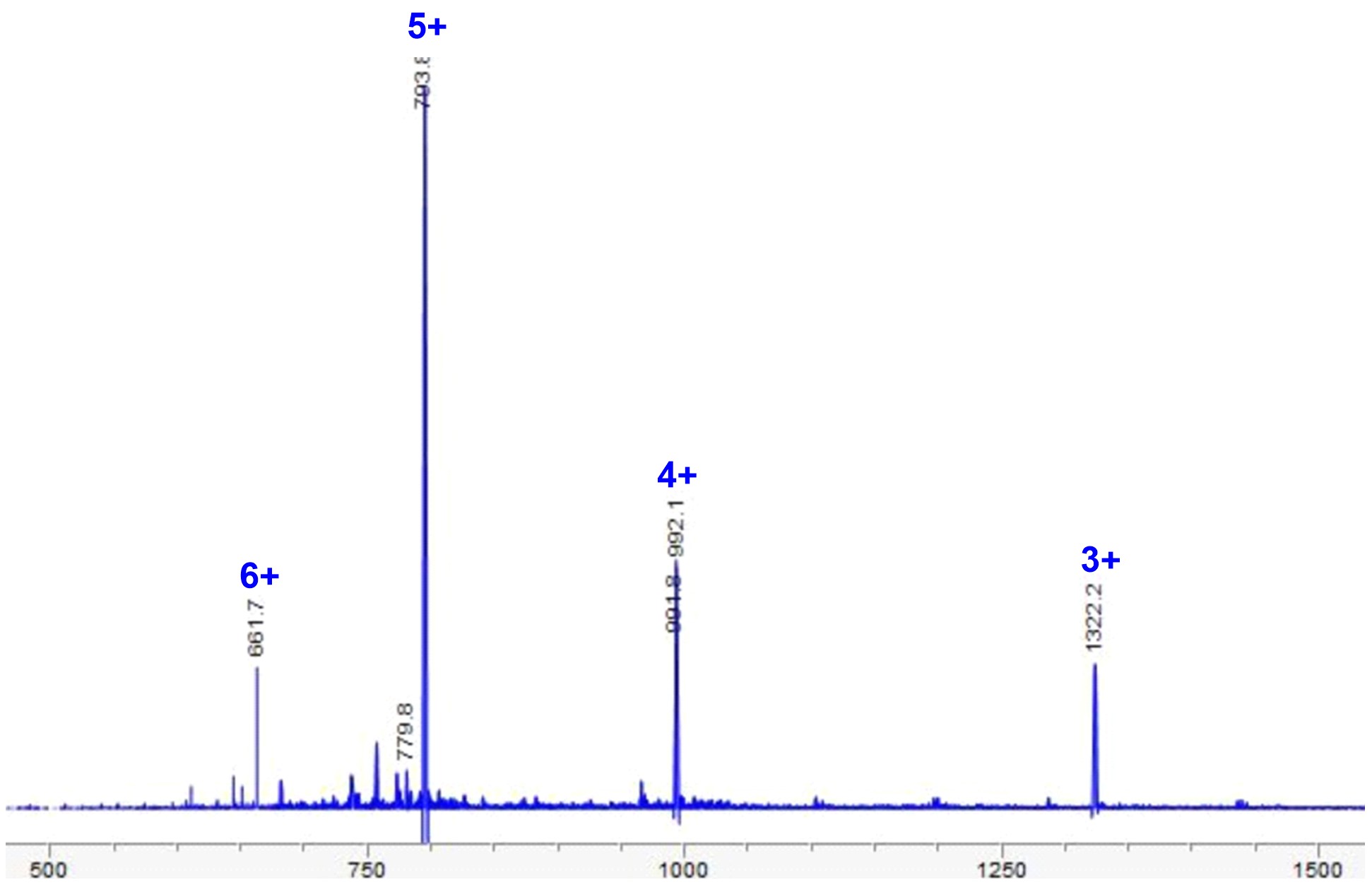


**Supplementary Figure 10.** (A) LC chromatogram (280 nm) and (B) MS spectrum of **6b**

A


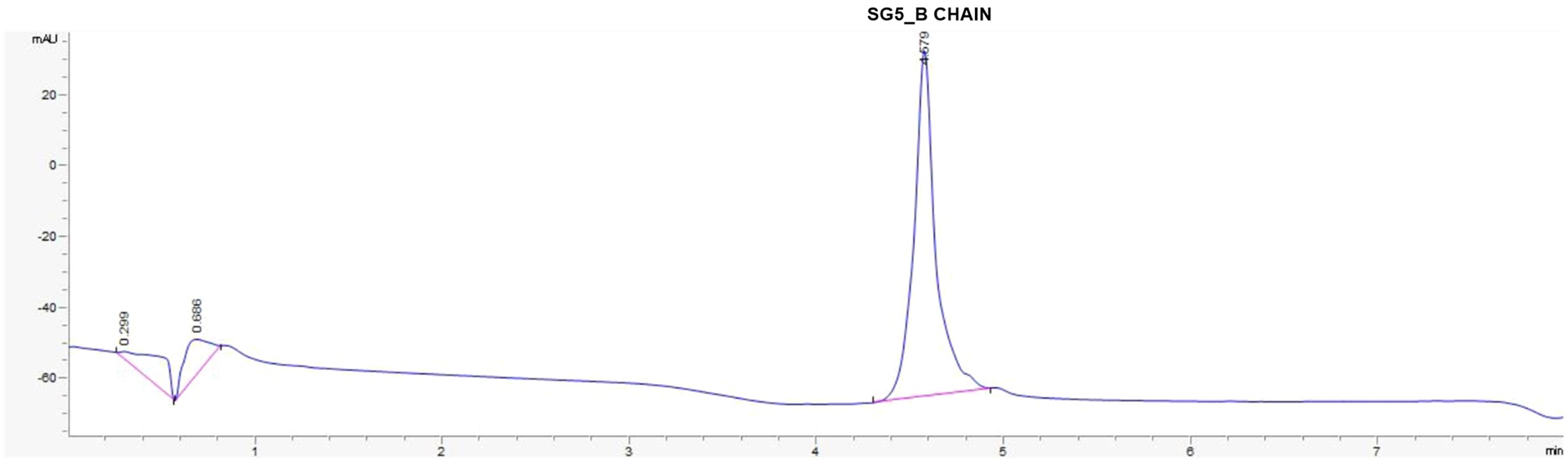


B


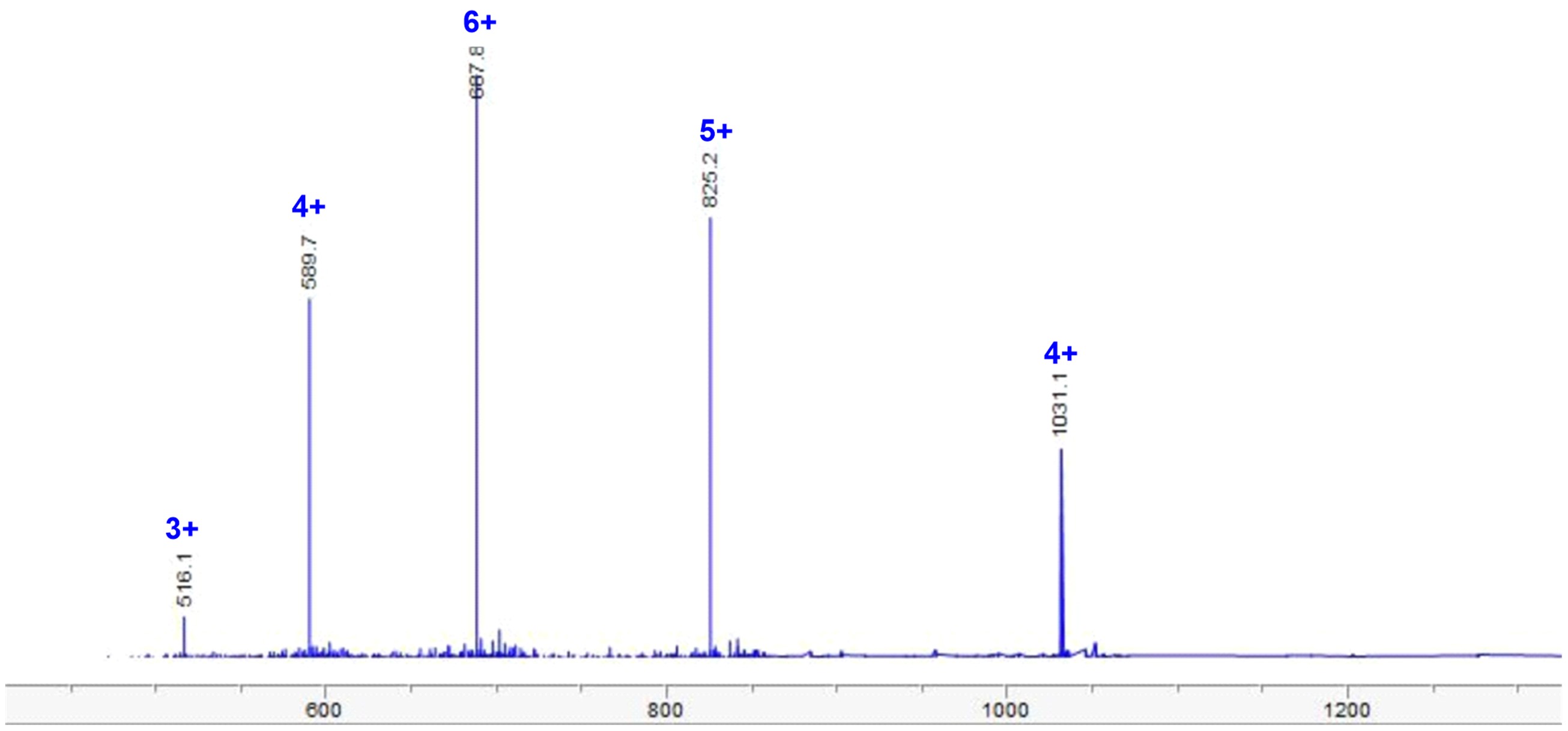


**Supplementary Figure 11.** (A) LC chromatogram (280 nm) and (B) MS spectrum of **6c**

A


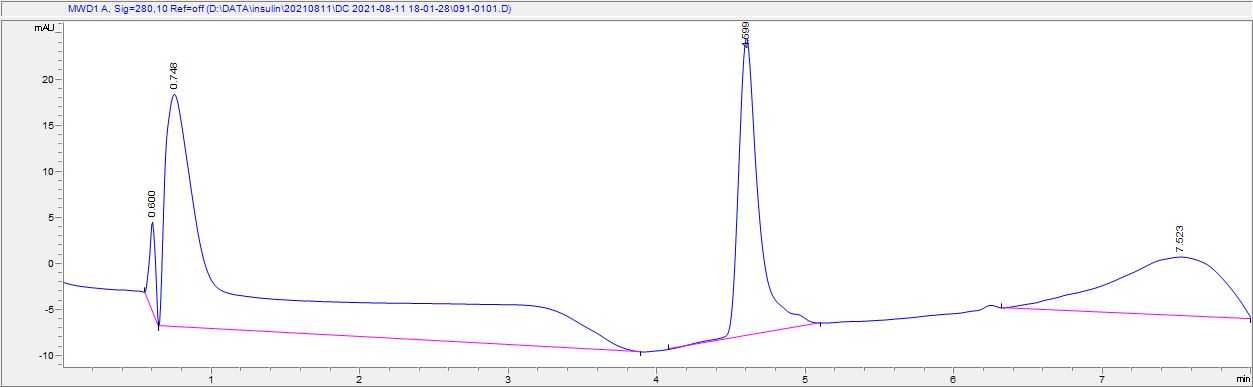


B


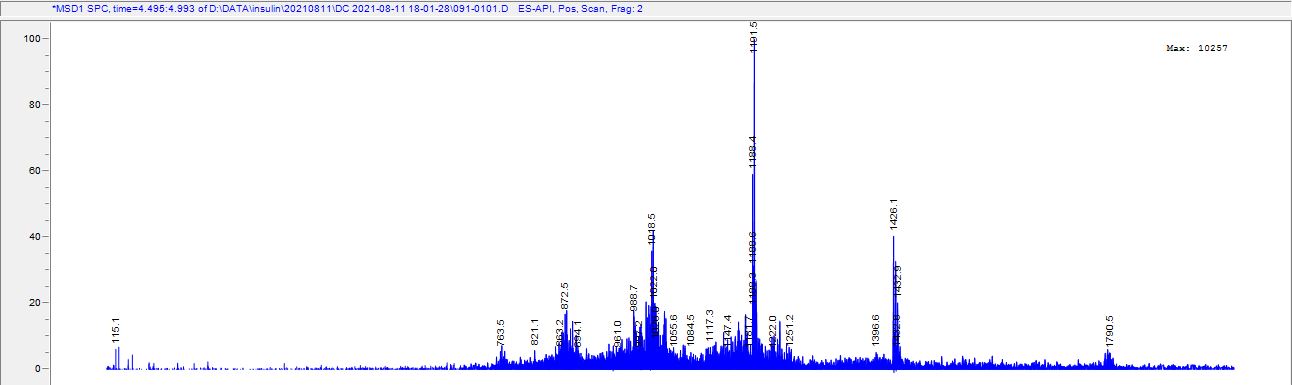


**Supplementary Figure 12.** (A) LC chromatogram (280 nm) and (B) MS spectrum of **7a**

A


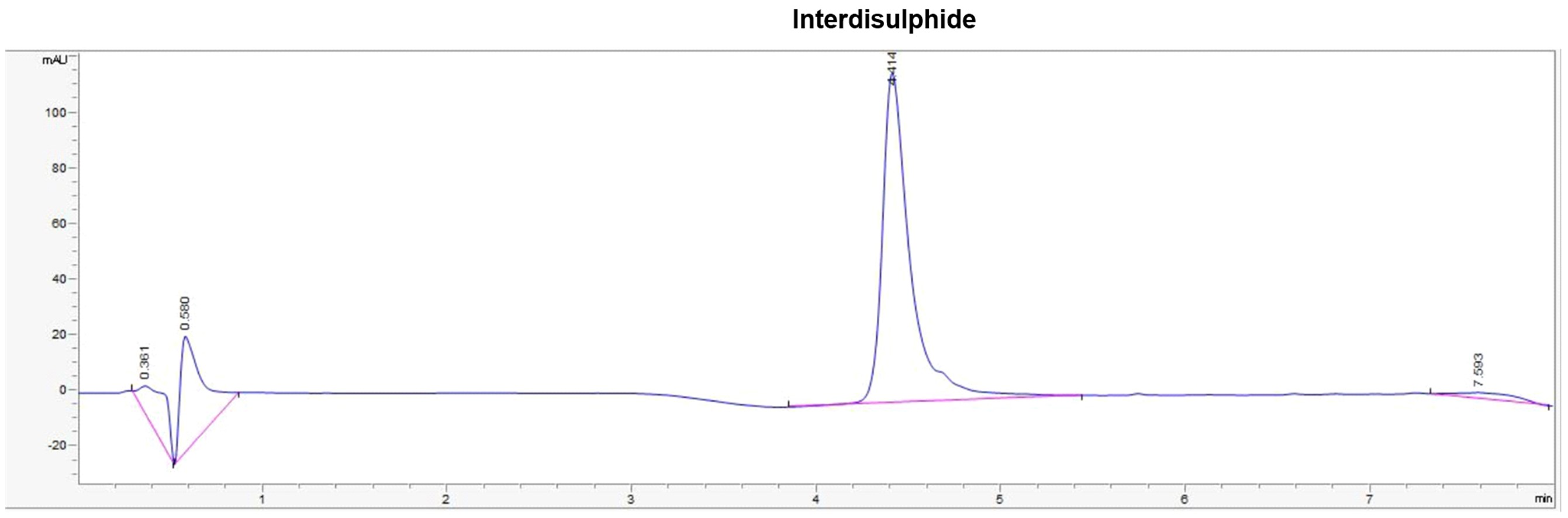


B


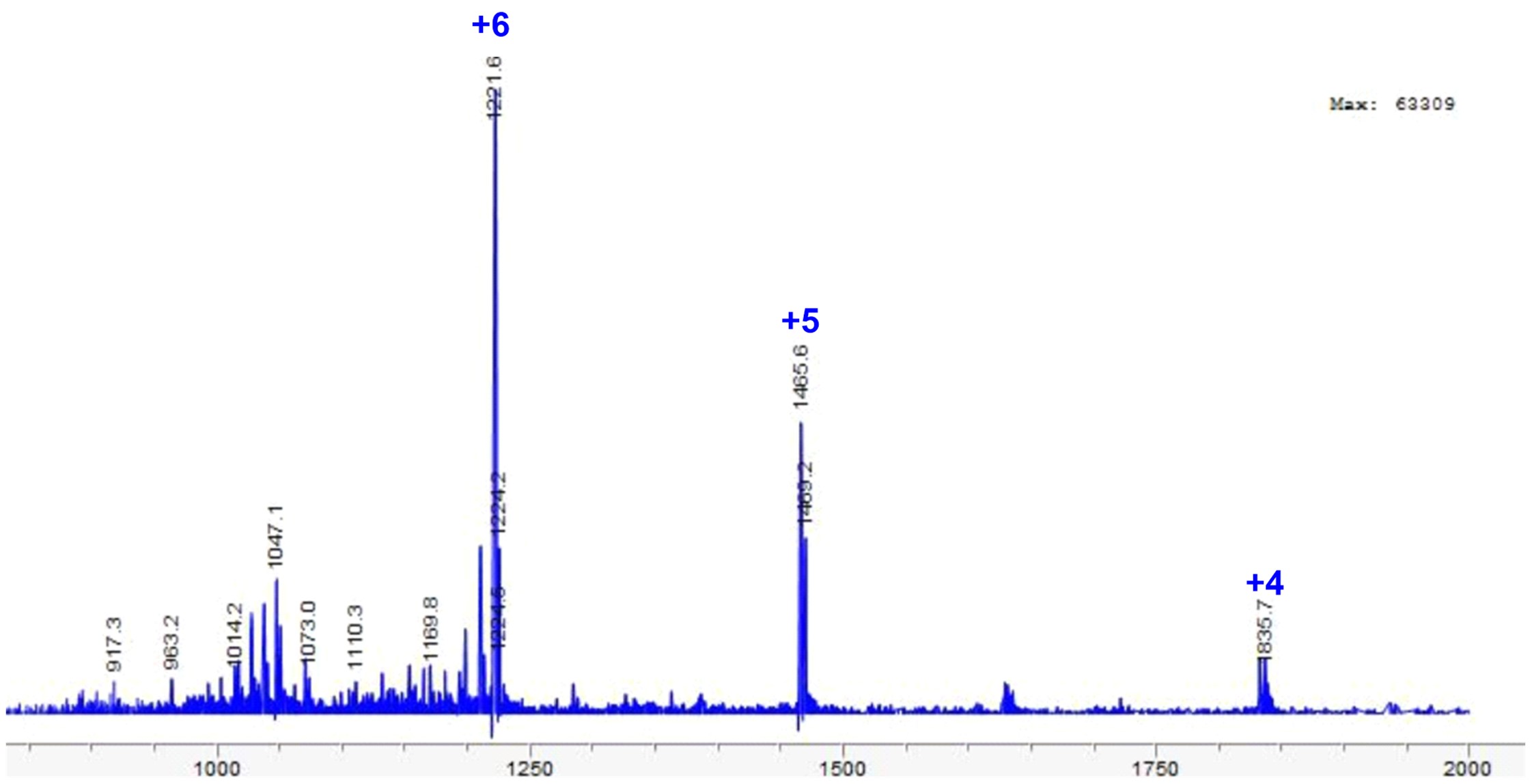


**Supplementary Figure 13.** (A) LC chromatogram (280 nm) and (B) MS spectrum of **7c**

A


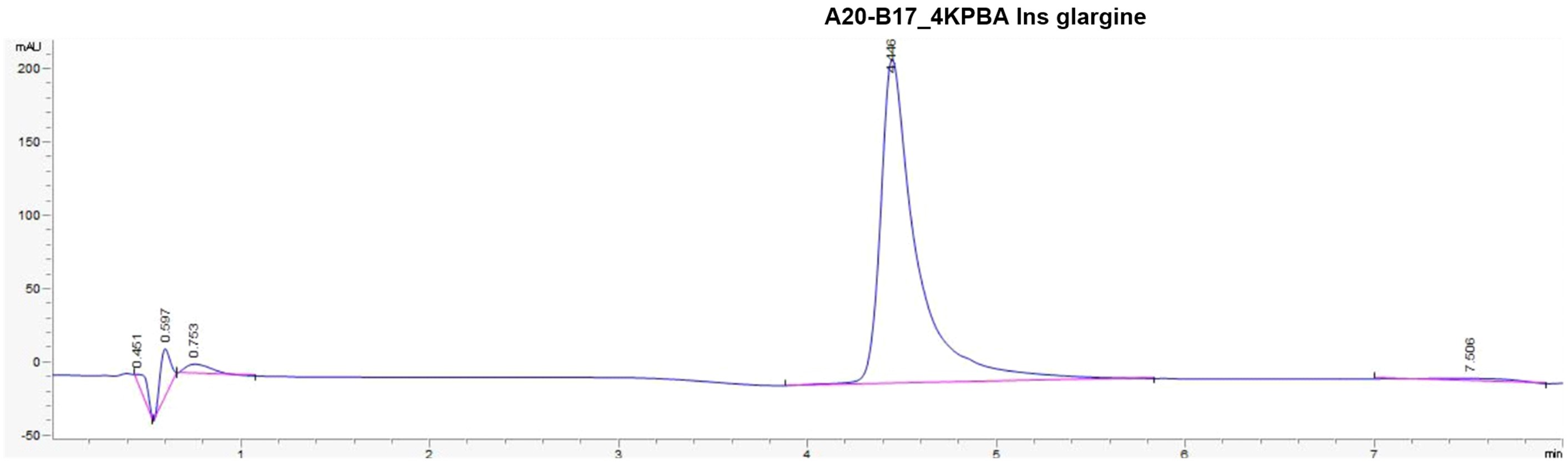


B


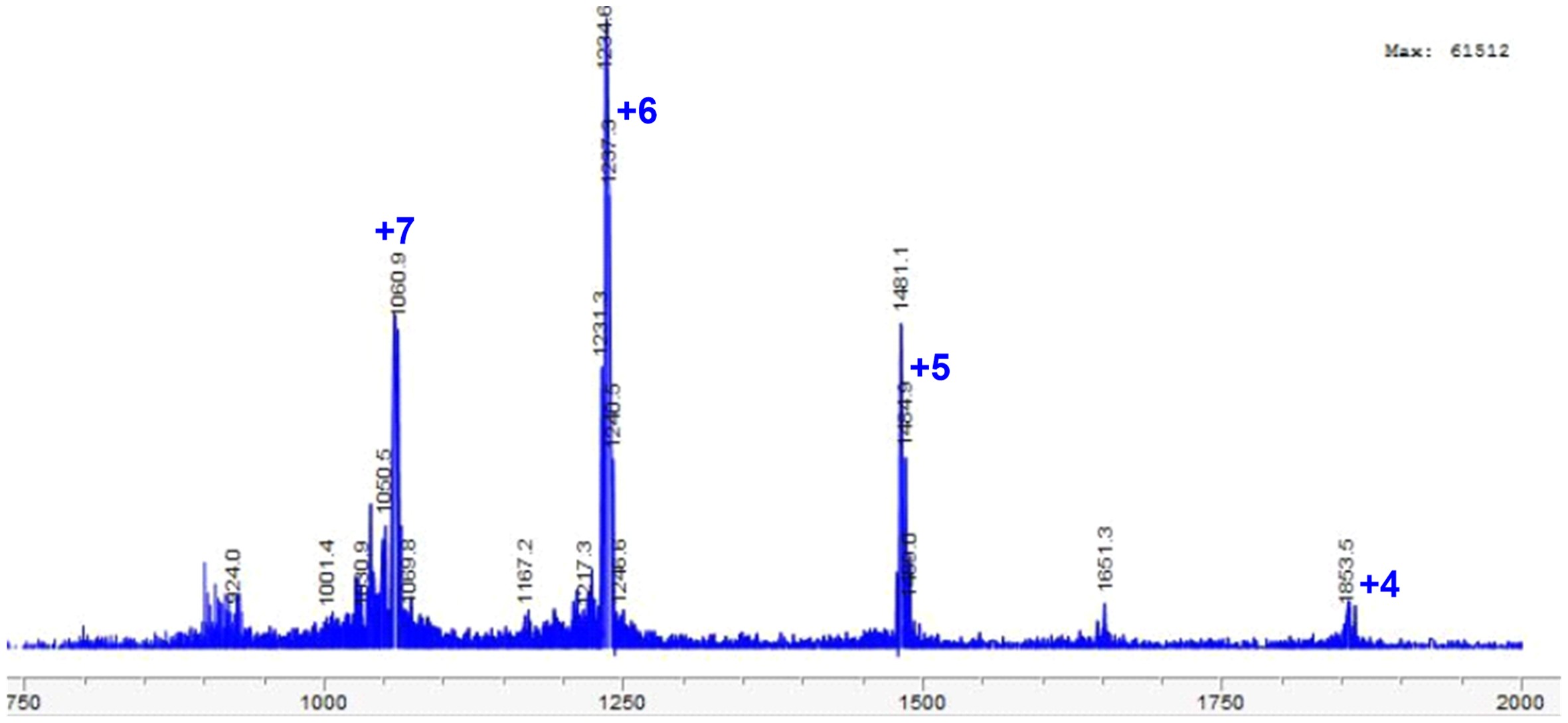


**Supplementary Figure 14.** (A) LC chromatogram (280 nm) and (B) MS spectrum of **7d**

A


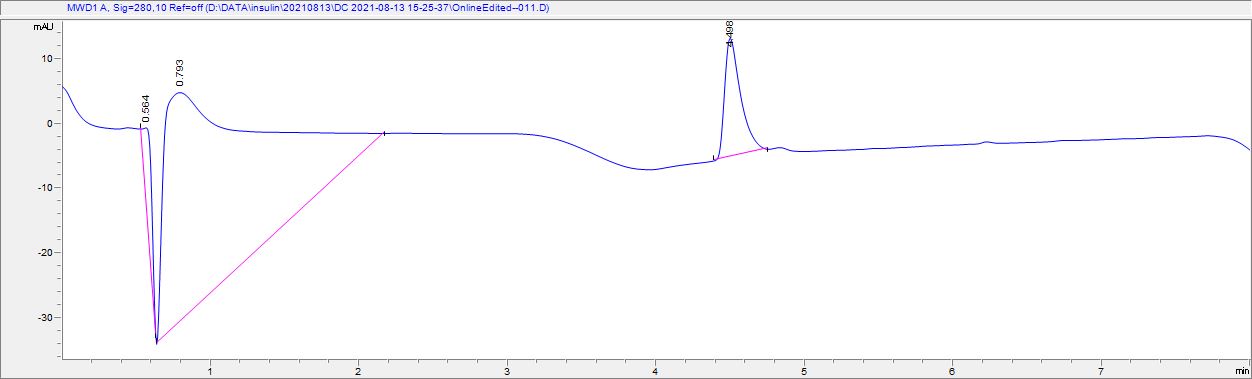


B


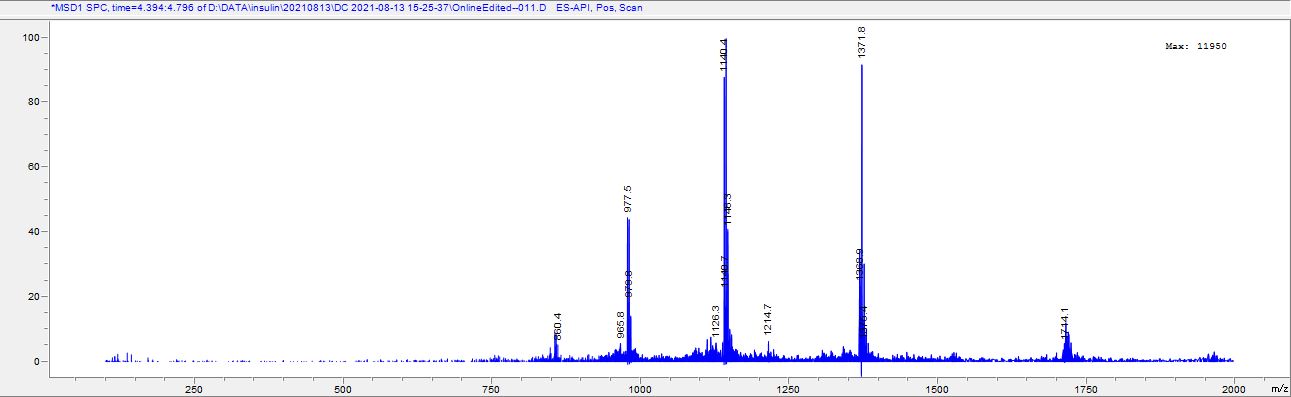


**Supplementary Figure 15.** (A) LC chromatogram (280 nm) and (B) MS spectrum of **8a**

A


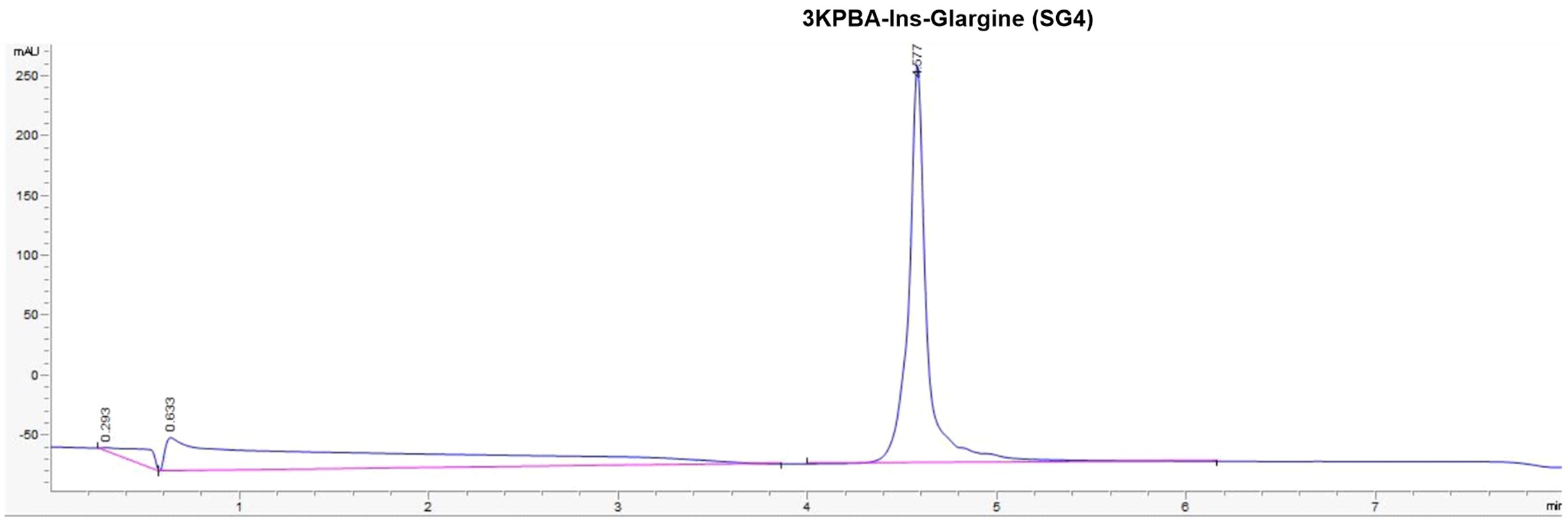


B


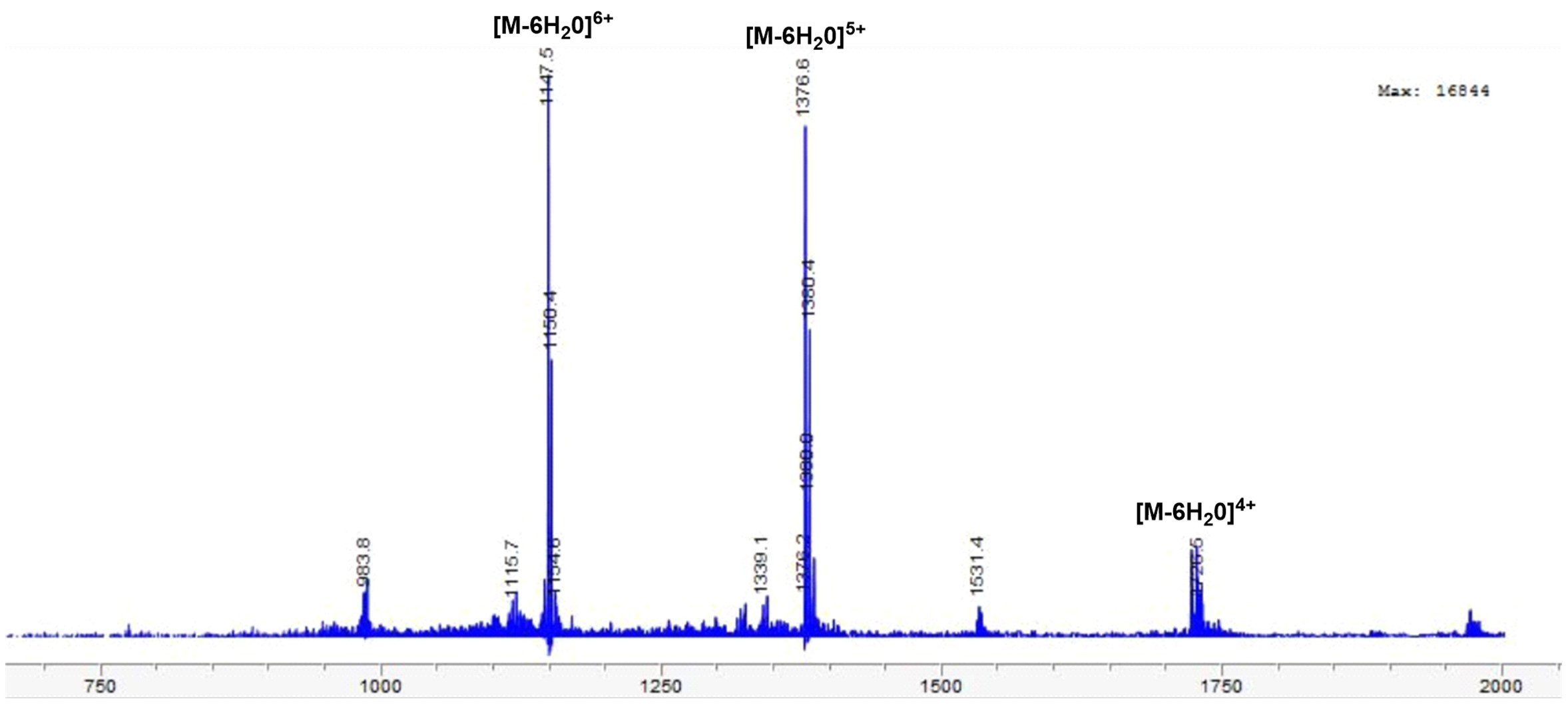


**Supplementary Figure 16.** (A) LC chromatogram (280 nm) and (B) MS spectrum of **8b**

A


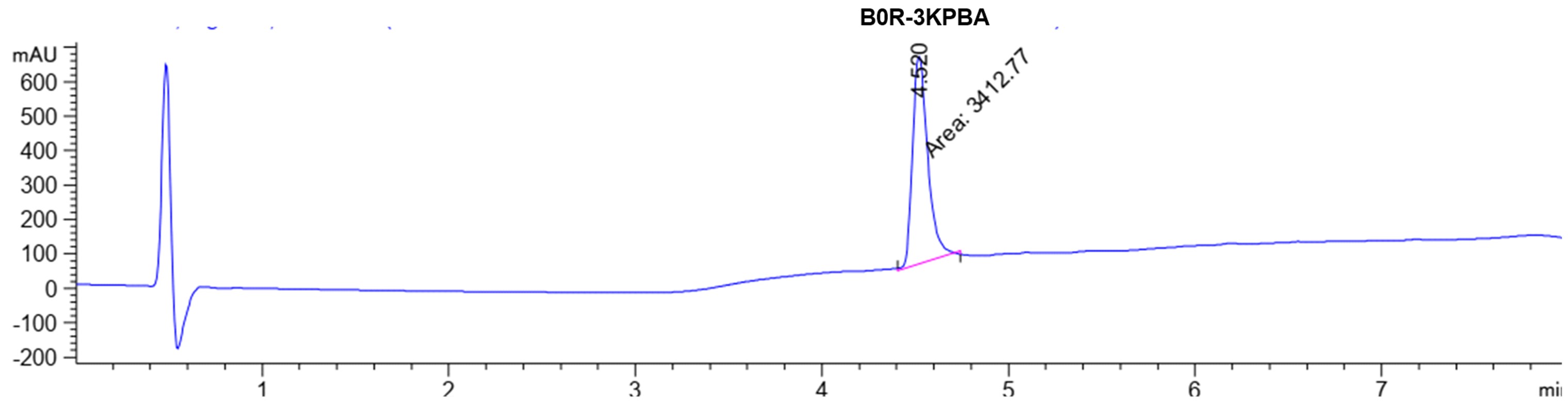


B


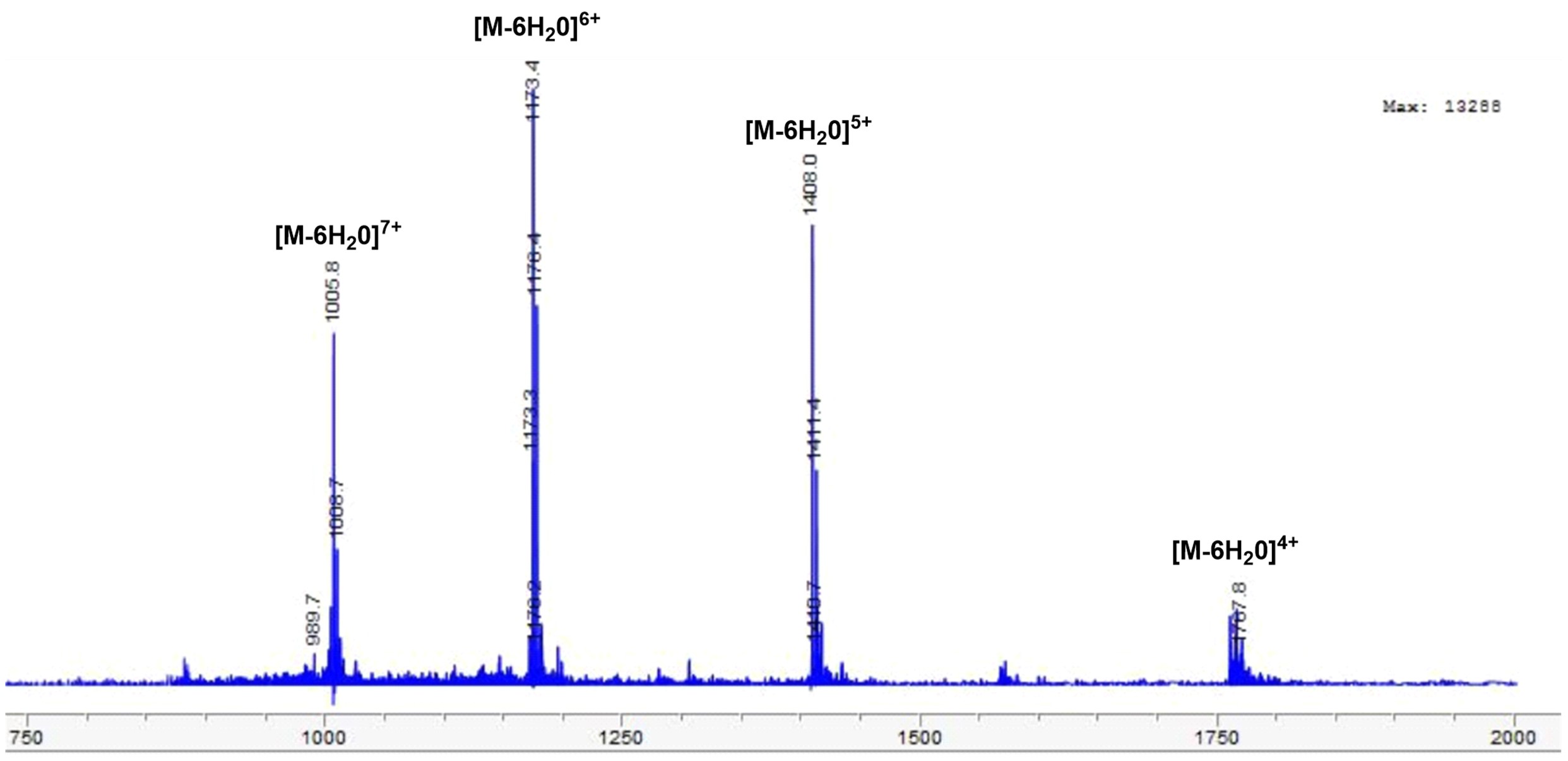


**Supplementary Figure 17.** (A) LC chromatogram (280 nm) and (B) MS spectrum of **8c**

A


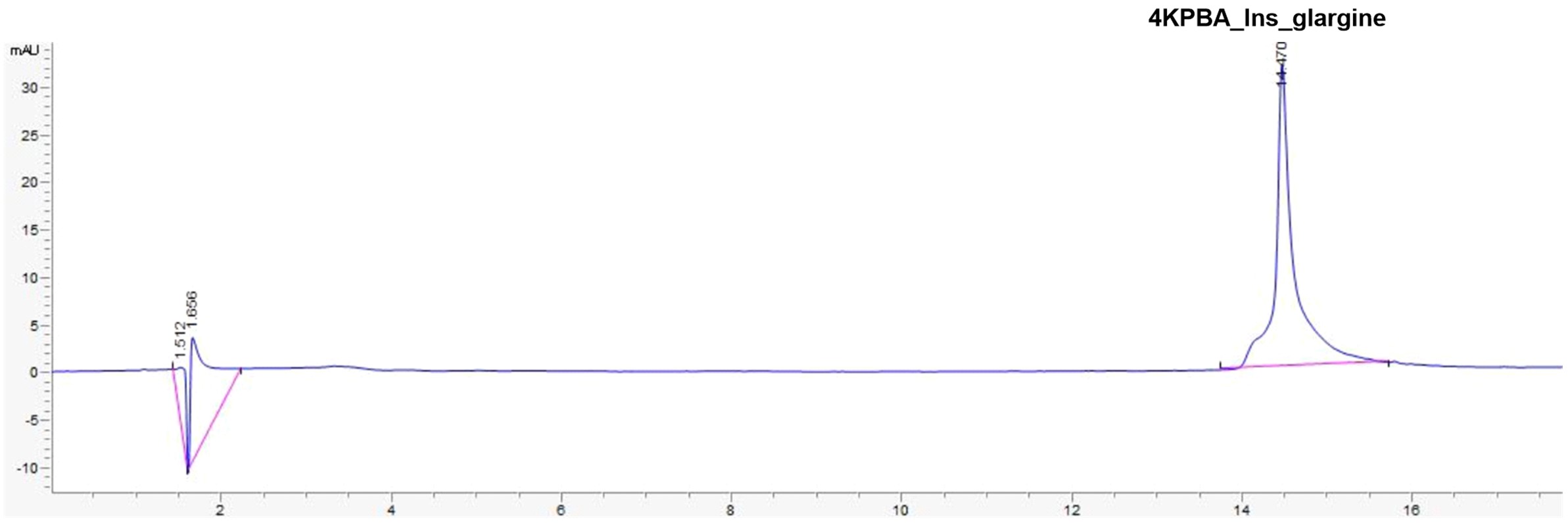


B


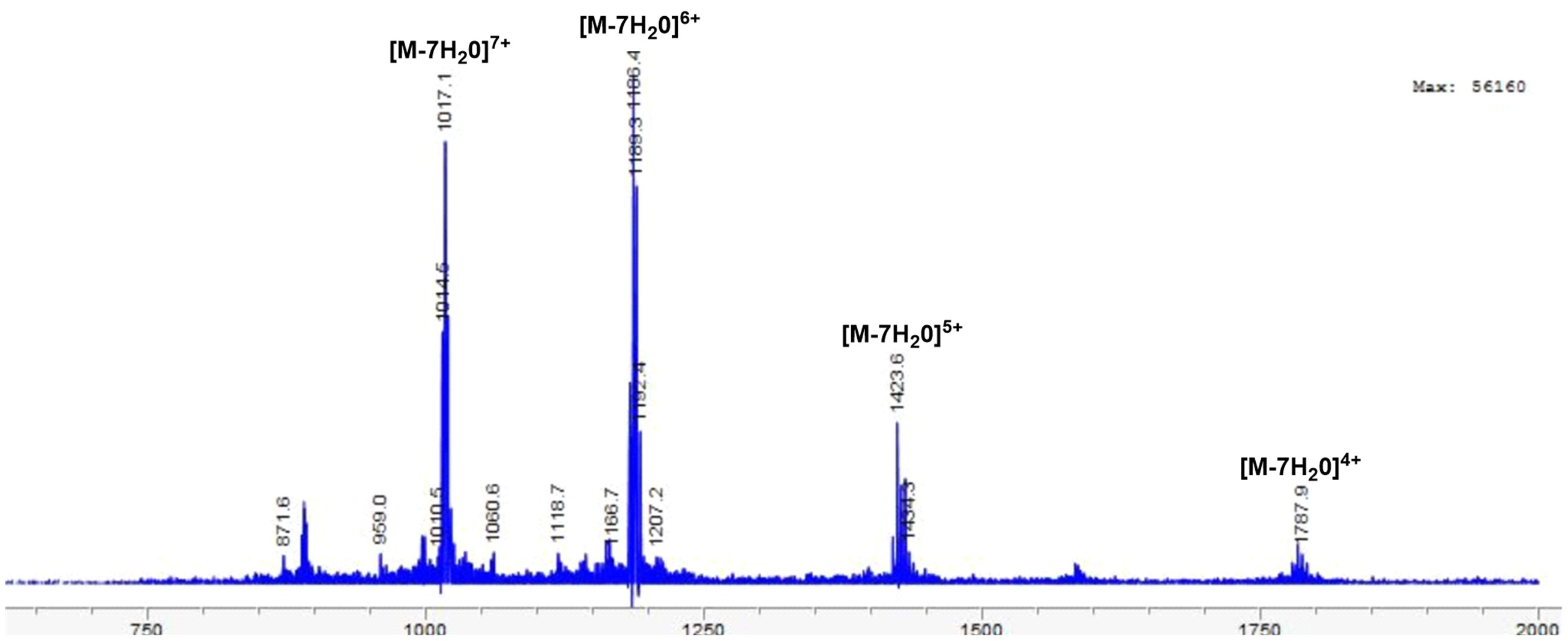


**Supplementary Figure 18.** (A) LC chromatogram (280 nm) and (B) MS spectrum of **8d**
